# Cryo-Structural Insights into Enzymatic Peptide Self-Assembly Driving Extrinsic Lytic Cell Death

**DOI:** 10.1021/jacs.5c23283

**Published:** 2026-03-24

**Authors:** Meihui Yi, Jiaqi Guo, Ayisha Zia, Wangbiao Guo, Shoichi Tachiyama, Gabriel Ashton-Rickardt, Weiyi Tan, Yuchen Qiao, Yinan Gong, Edward H. Egelman, Jun Liu, Fengbin Wang, Bing Xu

**Affiliations:** 1 Department of Chemistry, 8244 Brandeis University, Waltham, Massachusetts 02454, United States; 2 Microbial Sciences Institute, 5755 Yale University, West Haven, Connecticut 06516, United States; 3 Department of Microbial Pathogenesis, Yale School of Medicine, New Haven, Connecticut 06536, United States; 4 Department of Biochemistry and Molecular Genetics, 9967 University of Alabama at Birmingham, Birmingham, Alabama 35233, United States; 5 Department of Biochemistry and Molecular Genetics, University of Virginia, Charlottesville, Virginia 22908, United States; 6 Department of Immunology, University of Pittsburgh and UPMC Hillman Cancer Center, Pittsburgh, Pennsylvania 15232, United States

## Abstract

Programmed lytic cell death, including pyroptosis and necroptosis, involves intracellular enzymes that form membrane-rupturing pores. Tumor-associated ectoenzymes such as alkaline phosphatase (ALP), however, offer the potential to initiate lytic death extrinsically. Here, we design a phospho-biphenyl-capped peptide precursor that is selectively dephosphorylated by ALP on cancer cell surfaces, triggering enzyme-instructed peptide self-assembly (EISA) into in situ peptide filaments. These supramolecular filaments physically breach the plasma membrane, overwhelm ESCRT-dependent membrane repair, and induce catastrophic calcium influx, cytoskeletal collapse, and organelle dysfunction. While cryo-EM uncovers 2.5–2.9 Å resolution details of ordered dimeric packing that underlies their mechanical rigidity and membrane-rupturing capability, cryo-electron tomography (cryo-ET) reveals the filament penetration of the plasma membrane in live cells. By reprogramming ALP from an immune checkpoint ectoenzyme into a pro-death catalyst, this work establishes a molecular mechanism linking enzymatic catalysis to supramolecular order and membrane failure. More broadly, it outlines a supramolecular chemical–biology framework in which enzyme-triggered assemblies function as programmable executors of cell death.

## Graphical abstract

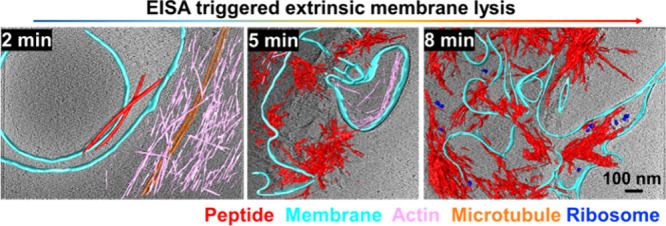

## Introduction

This article reports the first cryo-EM/ET mechanistic elucidation of extrinsic lytic cell death due to enzymatic self-assembly of small molecules.^1–4^ Programmed lytic cell death, typified by pyroptosis and necroptosis, represents a terminal event in which intracellular enzymes drive self-assembly of pore-forming proteins that rupture the plasma membrane from within. In pyroptosis, caspases cleave gasdermin D (GSDMD) to enable pore formation by self-assembled GSDMD oligomers.^5–7^ In necroptosis, receptor-interacting protein kinase 3 (RIPK3) phosphorylates mixed lineage kinase domain like pseudokinase (MLKL), triggering its oligomerization and subsequent membrane rupture.^8,9^ Together, these mechanisms illustrate how enzyme catalysis can direct molecular self-assembly to convert chemical energy into a mechanical event that dismantles cellular integrity. To survive such assaults, mammalian cells have evolved robust plasma membrane repair (PMR) mechanisms that rapidly restore membrane integrity. Endosomal sorting complex required for transport (ESCRT)-dependent ectocytosis, powered by the actin cytoskeleton and ATPases, excises damaged membrane segments to prevent lethal rupture.^10–14^ Tumor cells exploit this machinery to resist cytotoxic T lymphocyte (CTL) attack: after perforin-mediated pore formation, ESCRT proteins rapidly seal the lesions, blocking granzyme entry and enabling immune evasion.^15^ This repair response also mitigates gasdermin- and MLKL-induced rupture,^7,16,17^ establishing PMR as a critical defense barrier against immune- or drug-induced lytic death.

Tumors in hypoxic environments add a further layer of resistance through adenosinergic immunosuppression, driven by the ectoenzymes CD73 and alkaline phosphatase (ALP).^18,19^ ALP hydrolyzes extracellular adenesine triphosphate (ATP) to adenosine, which suppresses immune activity via A_2_A receptor signaling. Because ALP is essential in normal tissues such as bone, liver, and placenta,^20,21^ systemic inhibition is not viable. An alternative strategy is to convert ALP overexpression from a protective mechanism into a liability, using its catalytic activity to trigger cell death selectively in ALP-rich tumor microenvironments. Short peptides offer a minimal yet programmable platform for such reengineering.^22^ Their modular structures enable precise control over self-assembly, enzymatic responsiveness, and biological function. The concept of enzyme-instructed self-assembly (EISA) harnesses enzymatic catalysis to drive in situ organization of molecular precursors into functional nanostructures.^1,23,24^ While EISA has enabled intracellular and pericellular assemblies that regulate cell fate and inhibit tumor growth,^2,25–28^ the molecular and structural basis of these processes has remained poorly understood, particularly the relationship between enzyme catalysis, supramolecular order, and membrane disruption.

Here, we introduce a phospho-biphenyl-capped peptide precursor (**1P**) that undergoes ALP-triggered dephosphorylation to generate *in situ* peptide filaments (iPFs) on the plasma membranes of cancer cells that overexpress ALP. These extracellularly assembled iPFs physically penetrate the membrane, overwhelming ESCRT-mediated PMR and triggering a cascade of calcium influx, charged multivesicular body protein 4B (CHMP4B) recruitment, cytoskeletal collapse, mitochondrial fission, and widespread organelle dysfunction. Together, these events delineate a previously unrecognized extrinsic lytic death pathway, mechanistically distinct from canonical intracellularly programmed forms of lytic cell death.

To resolve this multiscale mechanism, we combine single-particle cryogenic electron microscopy (cryo-EM) and cryo-electron tomography (cryo-ET) to determine the 2.5–2.9 Å structures of iPFs and to visualize the spatiotemporal progression of iPFs assembly in intact cells. While cryo-EM reveals a recurring dimeric packing motif that accounts for the rigidity and membrane-breaching activity of iPFs, cryo-ET captures iPF emergence, membrane penetration, ectosome formation, and subsequent plasma membrane breakdown. Integrated with imaging and biochemical analyses, these complementary approaches establish a cryo-structural insights that link enzymatic catalysis to supramolecular assembly and, ultimately, to physical membrane failure (Figure 1b). By reprogramming ALP from an immune checkpoint enzyme^18^ into a pro-death catalyst, this work defines a chemical mechanism of extrinsic lytic cell death. Conceptually, it expands EISA from an intracellular phenomenon^24^ to a membrane-targeted, enzyme-triggered execution strategy. More broadly, this study establishes a supramolecular chemical biology framework for exploiting tumor-associated enzyme activity to control cell fate through the mechanics of self-assembly on cell membrane.

**Figure 1:**
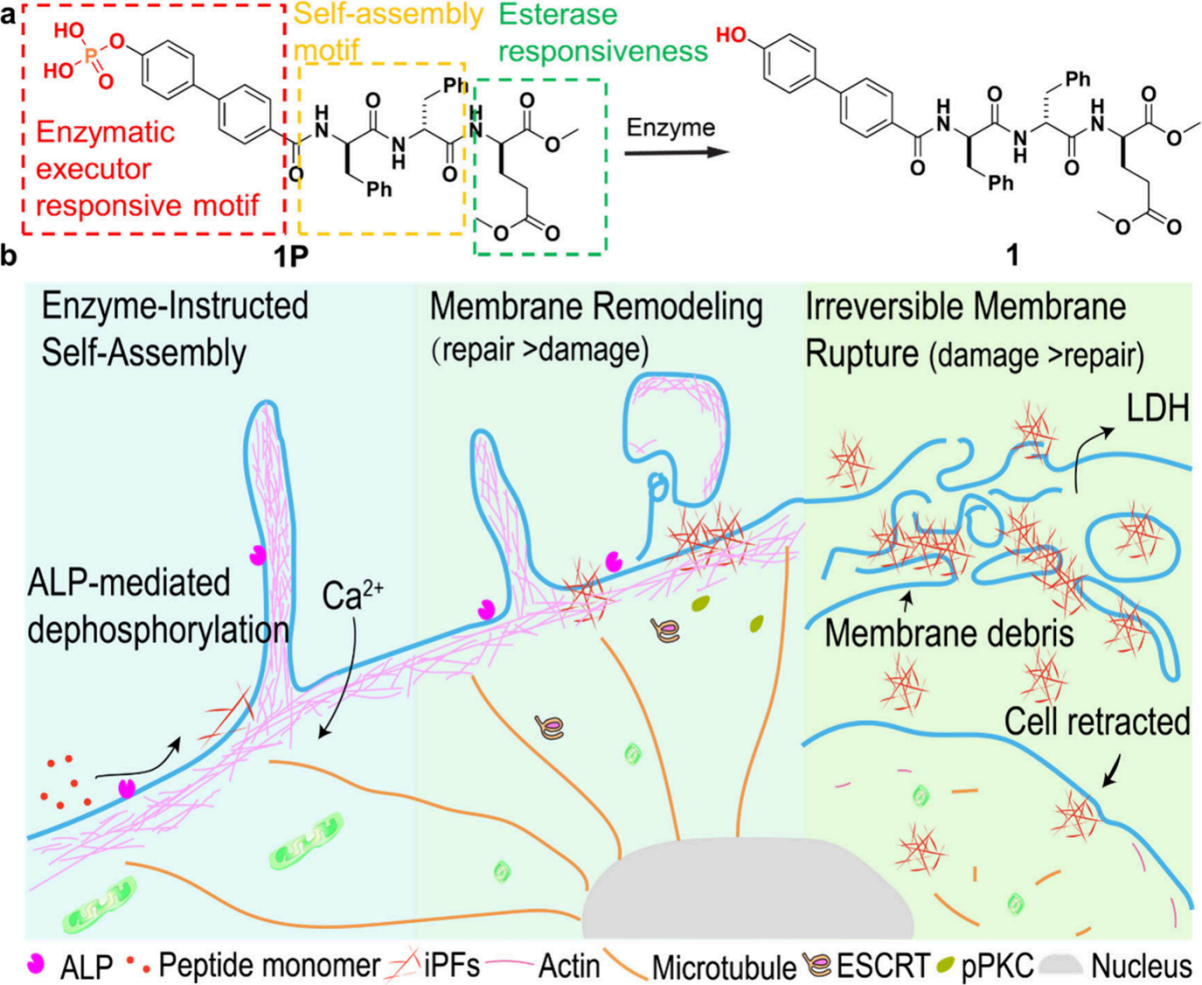
Enzymatic self-assembly drives extrinsic lytic cell death. (a) Molecular structures of **1P** and its dephosphorylated product, 1. (b) ALP catalyzes dephosphorylation of peptide precursors and the progressive plasma membrane damage.

## Results and Discussion

### ALP Catalyzes Peptide Self-Assembly into Filaments

ALP, one of earliest recognized oncodevelopmental markers,^29^ is an ectoenzyme frequently overexpressed in osteosarcoma and other tumors.^30,31^ Building on the concept of EISA,^1^ we designed a phospho-biphenyl-capped peptide (**1P**) as an ALP substrate capable of forming iPFs upon dephosphorylation (Figure 1a). The design couples three functional motifs: a phospho-biphenyl (pBP) group^32^ serving as both enzymatic executor responsive motif and aromatic assembly unit; a di-d-phenylalanine backbone (ff) imparting β-sheet-forming propensity;^33^ and a d-glutamate dimethyl ester terminus tuning solubility^34^ and showing esterase responsiveness. Dephosphorylation of **1P** by ALP converts it to **1**, a less soluble species that readily self-assembles into higher-order nanostructures for membrane remodeling. This transition lowers the critical aggregation concentration (CAC) from 420 μM for **1P** to 33 μM for **1** (Figure 2a), demonstrating that ALP catalysis directly drives self-assembly by reducing peptide solubility. Consistent with this, ALP treatment rapidly transforms **1P** (0.5 wt %) into a hydrogel within 24 h (Figure 2b).

**Figure 2:**
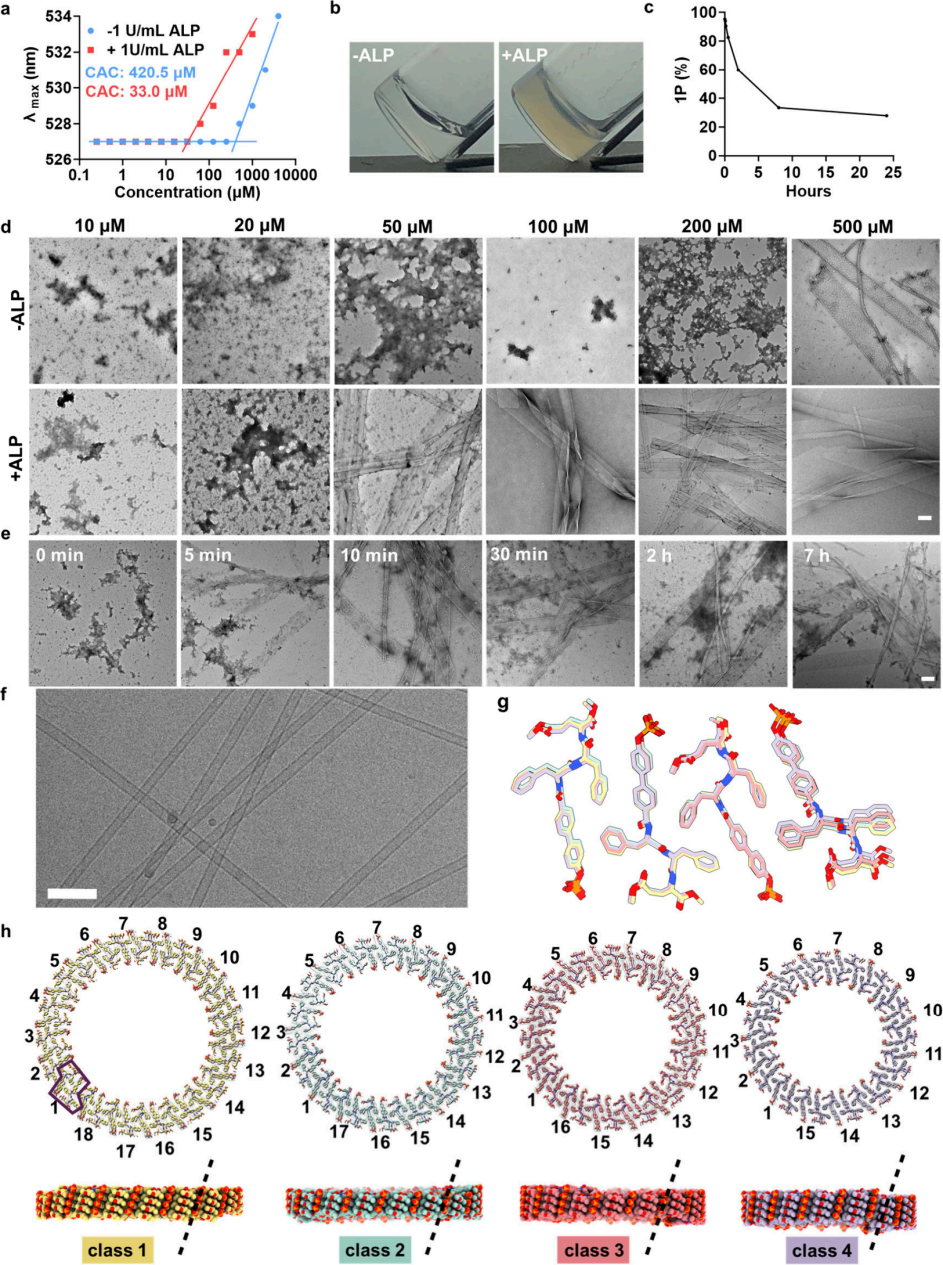
ALP-catalyzed self-assembly of **1P** into iPFs. (a) CAC of **1P** before and after ALP treatment in PBS buffer at pH 7.4. (b) Gel formation of **1P** (0.5 wt %) before and after ALP treatment for 24 h. (c) Time-dependent dephosphorylation of **1P** (100 μM) in Saos-2 cell lysate. (d) TEM images of **1P** before and after 24 h of ALP treatment in PBS (pH 7.4). (e) Time-resolved TEM images of **1P** (50 μM) showing the emergence of iPFs after ALP addition. Scale bar, 100 nm. [ALP] = 1 U/mL. (f) Cryo-EM images of self-assembled **1P** filaments. Scale bar, 50 nm. (g) Structural model showing two **1P** dimers that form the basic repeating unit of the nanotube. (h) Top views of reconstructed **1P** nanotubes with varying diameters, illustrating structural plasticity among distinct helical assemblies.

Incubation of **1P** with the lysate of Saos-2 cells, which express high levels of ALP, results in about 80% dephosphorylation within 24 h (Figure 2c), confirming that endogenous ALP efficiently processes the peptide. Negative-stained transmission electron microscopy (TEM) revealed distinct morphological evolution during this enzymatic transformation. Before ALP addition, **1P** remained soluble or formed occasional aggregates below 500 μM; at 500 μM, it assembled into nanofibers and nanosheets (Figure 2d). Followed by ALP treatment of **1P**, **1** predominantly self-assembled into nanosheets at concentrations ≥ 50 μM, consistent with its CAC. Time-resolved TEM images showed that fibrillar structures emerged within 5 min after ALP addition (Figure 2e), indicating rapid ALP-triggered self-assembly. Together, these results demonstrate that ALP catalyzes a dephosphorylation-to-assembly transformation, converting soluble precursors into filamentous supramolecular structures. This reaction exemplifies how enzyme activity can serve as a biochemical trigger for spatially localized self-organization, the central principle underlying EISA, and establishes the molecular basis for the extrinsic lytic mechanism.

### Cryo-EM Resolves the Molecular Architecture of Peptide Filaments

Interestingly, we observed nanotube formation for both **1P** and **1** (Figure 2d, e). At high peptide concentrations, **1P** predominantly formed nanotubes, while **1** primarily yielded nanosheets, with nanotubes appearing only sporadically. To gain structural insight into peptide self-assembly, we used cryo-EM to determine their atomistic structures. Because in situ conditions likely reach higher local peptide concentrations than in vitro PBS due to enzymatic dephosphorylation and reduced diffusion, it is probable that the nanosheet morphology observed in cells (vide infra) evolves from initial nanotube structures. Thus, we focused on nanotube morphology of **1P** at pH 5.6. We chose mildly acidic conditions (pH 5.6) because our previous findings that nanotubes formed from a phosphorylated precursor under acidic pH exhibit nearly identical high-resolution structures to those of dephosphorylated nanotubes generated at physiological pH.^35^ Thus, here, we use the same condition to obtain homogeneous assemblies suitable for cryo-EM analysis, and it is expected to capture structural features relevant to the nonclustered enzyme-triggered assemblies. While the cryo-EM structure of **1P** is relevant to individual filaments of **1**, time-resolved cryo-ET offers the ultrastructural details during the early interactions of **1P** and **1** with cells in their native context.

Under cryo-EM, we observed nanotubes with diameters ranging from 10 to 14 nm (Figure 2f), displaying morphologies consistent with those observed under negative staining. One-dimensional projections derived from 2D averages revealed distinct diameters separated by ∼0.7 nm, allowing us to sort the classes for helical symmetry determination and high-resolution 3D reconstruction. The final reconstructions of four distinct diameters reached resolutions of 2.9, 2.8, 2.6, and 2.5 Å, respectively (Table S1). The top views of these nanotubes revealed that all four share a similar building block: a **1P** dimer (Figure 2g). The interfaces between building blocks among the four different nanotubes were nearly identical, with an Root-Mean-Square Deviation (RMSD) of 0.3–0.5 Å when aligning the backbones of two **1P** dimers across the four nanotubes (Figure 2h). The peptide–peptide interface along the helical axis formed a slightly right-handed twisted protofilament. Because the study employed d-peptides, the corresponding l-peptide would produce left-handed protofilaments, consistent with recent cryo-EM studies of protein and peptide amyloids, which report that nearly all cross-β filaments adopt left-handed architectures.^36–38^ Overall, the nanotubes detected in cryo-EM consist of the same **1P** dimer building block, exhibiting structural plasticity that accommodates slightly different interfaces. This plasticity results in varying numbers of protofilaments per nanotube, thereby generating distinct diameters (Figure 2h). Such quasi-equivalence phenomena^39^ have been reported in other helical polymers recently, including archaeal spindle-shaped virus SMV1 ^40^ and possibly bacterial gas vesicles.^41^

### ALP Expression Dictates the Selectivity of Peptide-Induced Cytotoxicity

Because ALP is an ectoenzyme anchored on the outer leaflet of the plasma membrane, we reasoned that ALP-triggered dephosphorylation of **1P** would generate iPFs directly at the cell surface, leading to localized self-assembly and selective cytotoxicity in ALP-rich cancer cells. We evaluate the cytotoxicity of **1P** across four cell lines differing in their phosphatase profiles: Saos-2 and SJSA-1, two osteosarcoma cell lines with high ALP expression; HS-5, a human marrow stromal cell line with normal ALP expression and high acid phosphatase (ACP) expression;^42^ and HepG-2, a hepatocyte cell line expressing both ALP and carboxylesterases.

**1P** potently inhibited proliferation of Saos-2 and SJSA-1 cells (GI_50_ ≈ 5 μM at 24 h), while showing minimal effect in HS-5 cells, underscoring its dependence on ALP activity (Figure 3a and Figure S1). Comparison of GI_50_ and GI_90_ at 72 h across all lines revealed a clear correlation between ALP expression and peptide sensitivity: the highest activity occurred in Saos-2, intermediate in SJSA-1, and low in HS-5 and HepG-2 (Figure 3b and Figure S2). The GI_50_ of **1P** is below the CAC of **1** because the CAC is measured under cell-free conditions, while in the crowded cellular environment ALP-rich membrane domains locally increase the effective concentration of **1** beyond its CAC, enabling cytotoxic assembly at lower incubation concentrations. To further verify enzyme dependence, we cotreated Saos-2 or SJSA-1 cells with **1P** and the ALP inhibitor 2,5-dimethoxy-N-(quinolin-3-yl)­benzamide (DQB),^43^ which rescued cell viability from ∼3% to 46% (Saos-2) and 70% (SJSA-1) (Figure 3c and Figure S3). The partial rescue in Saos-2 is consistent with that Saos-2 also expresses the ALPP2 isoform^44^ and DQB is a selective inhibitor for ALPL.^45^ In addition, we engineered the surface of HS-5 cell with exogenous alkaline phosphatase through antigen–antibody binding strategy. The increased ALP density markedly enhanced the cytotoxicity of **1P** toward HS-5 cells (Figure S4). Together with the inhibitory experiments, these results highlight the pivotal role of ALP in mediating the activity of **1P** and the potential of targeting different cells through engineering cell surface. In HepG-2 cells, **1P** displayed weaker cytotoxicity, likely due to hydrolysis of the d-glutamate dimethyl ester terminus by carboxylesterases,^46^ which was confirmed by the enhanced cytotoxicity of **1P** against HepG-2 with the cotreatment of bis­(4-nitrophenyl) phosphate (BNPP) (Figure S5).

**Figure 3:**
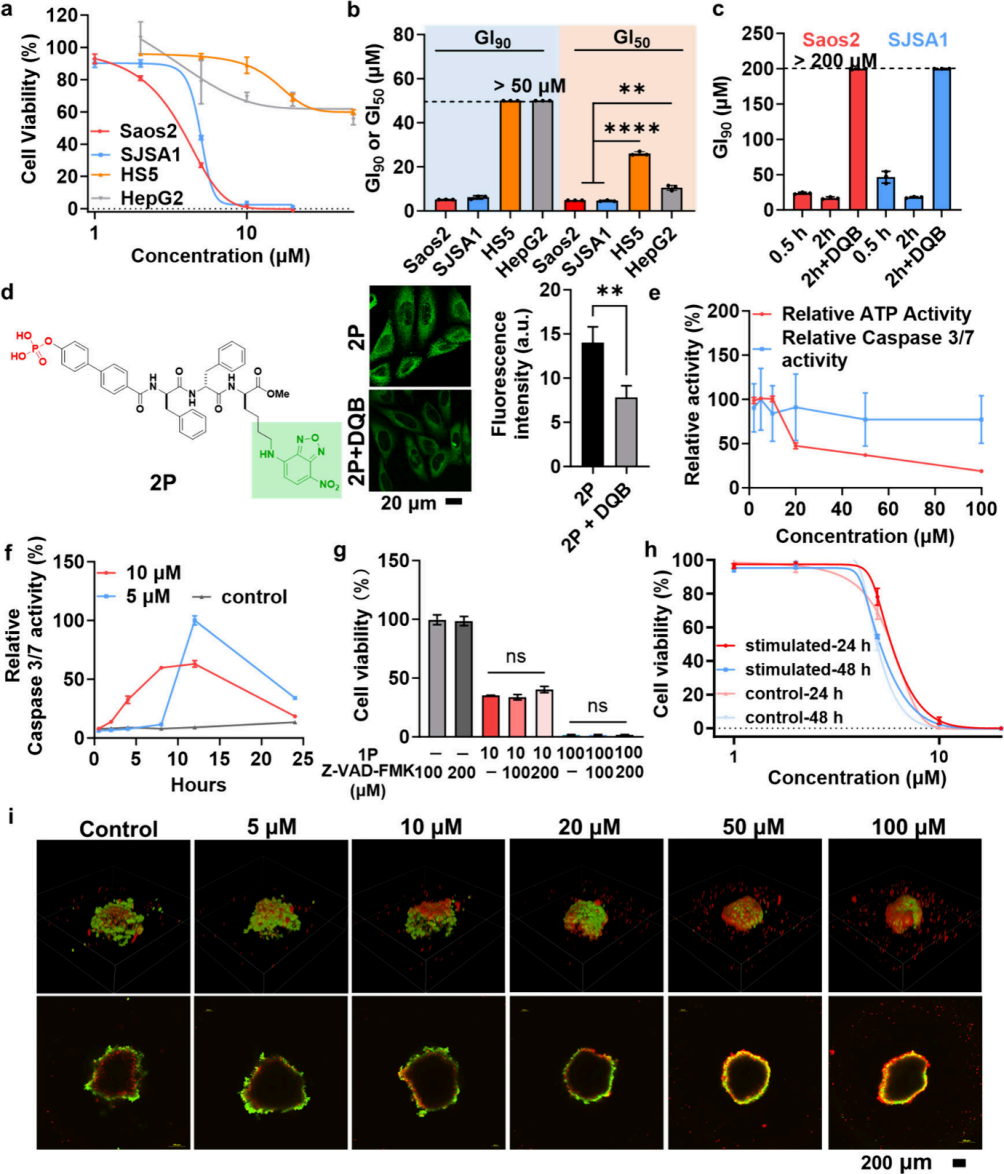
ALP expression governs **1P**-induced cytotoxicity. (a) Cell viability of Saos-2, SJSA-1, HepG-2, and HS-5 cells after 24 h treatment with **1P**. (b) GI_50_ and GI_90_ values of **1P** against Saos-2, SJSA-1, HepG-2, and HS-5 cells after 72 h. Data are shown as mean ± SD (*n* = 3). (c) Cell viability of Saos-2 or SJSA-1 treated with **1P** at 0.5, 2, or 2 h with DQB. (d) Molecular structure and cellular uptake of **2P** (20 μM) by Saos-2 with or without the co-incubation of DQB (20 μM), along with quantitative analysis. Data are shown as mean ± SD (*n* = 6). (e) Relative ATP activity and caspase 3/7 activity of Saos-2 cells with **1P** (2, 5, 10, 20, 50, or 100 μM) for 30 min. (f) Time-dependent caspase-3/7 activation in Saos-2 cells treated with **1P** (0, 5, or 10 μM). (g) Cell viability of Saos-2 cells co-incubated with **1P** and the pan-caspase inhibitor Z-VAD-FMK. Data are shown as mean ± SD (*n* = 3). (h) Cell viability of Saos-2 cells treated with **1P** for 24 or 48 h, including stimulated and nonstimulated conditions. (i) Live/dead confocal images of Saos-2 spheroids treated with **1P** (0, 10, 20, 50, or 100 μM) for 24 h. Statistical significance was determined using an unpaired two-tailed Student’s *t* test. *p < 0.05, **p < 0.01, ***p < 0.001, ****p < 0.0001.

To examine the ALP-dependent behavior of the peptide in cells, we synthesized a fluorescent analogue **2P** (Figure 3d). Live-cell imaging after 30 min treatment showed punctate intracellular fluorescence, suggesting that a fraction of the peptide (or its dephosphorylated assemblies) can be internalized following ALP-triggered activation at the cell surface. In contrast, cotreatment with the ALP inhibitor DQB resulted in a much weaker and more diffuse fluorescence signal, supporting the critical role of ALP activity in promoting peptide dephosphorylation and subsequent assembly associated cellular uptake.^47^ Bright-field images of DQB-treated cells (Figure S6) show no black puncta within the cells or dark regions on the plasma membrane, supporting that cellular entry of **2P**/**2** results from ALP-catalyzed dephosphorylation. Caspase-3/7 activity and ATP assays revealed rapid, nonapoptotic cytotoxicity. Upon exposure to **1P** (2–100 μM) for 30 min, cellular ATP levels decreased immediately, whereas caspase-3/7 activity remained largely unchanged (Figure 3e). Short-term treatment (≤100 μM, 30 min) did not activate caspase-3/7, whereas prolonged incubation (2–8 h) induced moderate caspase activity (Figure 3f), suggesting that early death is caspase-independent but may be followed by secondary apoptotic responses. Consistent with this, the pan-caspase inhibitor Z-VAD-FMK failed to rescue viability (Figure 3g), confirming that **1P** induces a primary, nonapoptotic death pathway.

Repeated-dose experiments showed no acquired resistance after ten treatment cycles (Figure 3h), highlighting the robustness of this supramolecular drug mechanism. In three-dimensional Saos-2 spheroids, **1P** produced extensive peripheral cell death within 24 h (Figure 3i), demonstrating that the enzyme-activated assembly retains efficacy in tumor-like microenvironments.

Together, these findings establish **1P** as an enzyme-responsive anticancer precursor that exploits endogenous ALP activity to trigger localized supramolecular self-assembly and rapid, caspase-independent lytic death in ALP overexpressing cells.

### Self-Assembled Filaments Breach Cancer Cell Membranes and Outpace Repair

Because ALP is displayed on the outer leaflet of the plasma membrane, its catalytic action occurs extracellularly, producing iPFs that accumulate along the cell surface. We hypothesized that these filaments physically disrupt the plasma membrane, triggering a cascade of plasma membrane damage (PMD) and PMR responses culminating in extrinsic lytic death. To test this hypothesis, we monitored real time changes in membrane integrity using a plasma membrane impermeable fluorescent dye at varying **1P** concentrations. At 10 μM **1P**, the dye remained extracellular after 30 min, and cells exhibited only slight shrinkage, indicating intact membranes (Figure 4a). At 50 μM, however, the dye rapidly entered cells and accumulated in the nucleus within 15 min, revealing acute, concentration dependent membrane disruption. These results suggest that a threshold concentration of ALP substrates is required to compromise membrane integrity in short time.

**Figure 4:**
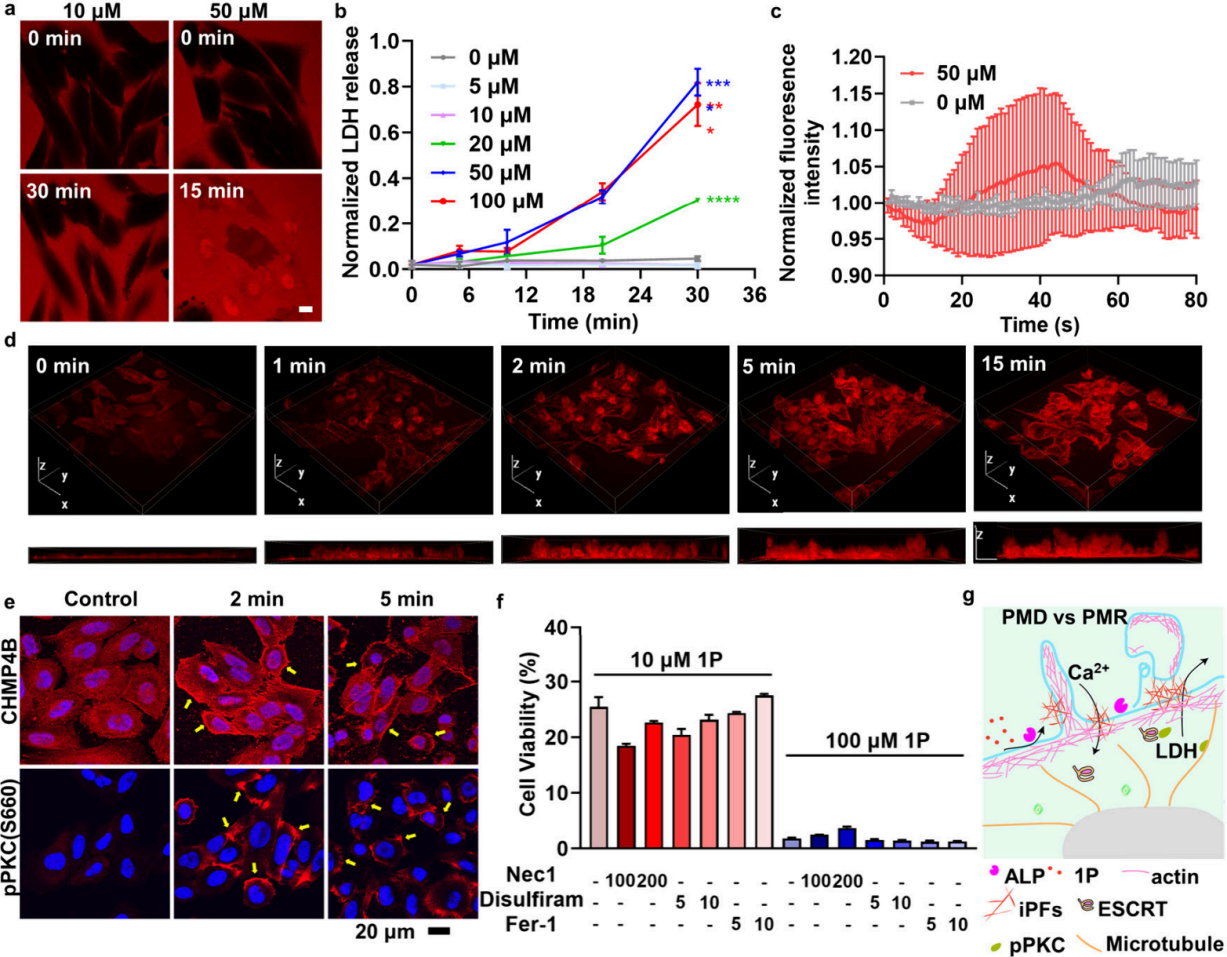
Self-assembled filaments breach the plasma membrane and overwhelm repair. (a) Fluorescence images showing plasma membrane rupture in Saos-2 cells treated with **1P** (10 or 50 μM), indicated by entry of the membrane-impermeable dye (red). (b) Quantification of lactate dehydrogenase (LDH) release from Saos-2 cells treated with **1P** (5–100 μM) for 30 min. (c) Intracellular calcium dynamics in Saos-2 cells with or without **1P** treatment over 80 s (*n* = 6). (d) Plasma membrane staining of Saos-2 cells treated with **1P** (100 μM) for indicated time points, showing progressive morphological collapse. (e) Plasma membrane translocation of CHMP4B and phosphorylated PKC­(S660) in Saos-2 cells following **1P** treatment (100 μM) at different time points. Red color, CHMP4B or phosphorylated PKC­(S660). Blue color, nucleus. (f) Cell viability of Saos-2 cells co-incubated with **1P** (10 or 100 μM) and inhibitors of necroptosis (Nec-1, 100 or 200 μM), pyroptosis (disulfiram, 5 or 10 μM), or ferroptosis (Fer-1, 5 or 10 μM) for 2 h. (g) Schematic illustration of the competition between PMD and PMR.

We next quantified membrane damage using lactate dehydrogenase (LDH) release, an indicator of plasma membrane rupture. LDH release remained minimal at low **1P** concentrations but increased markedly with higher concentrations and prolonged incubation (Figure 4b), confirming dose- and time-dependent membrane permeabilization caused by iPFs. Because calcium influx is an early hallmark of PMD,^48^ we monitored intracellular calcium level using a fluorescent calcium dye. As shown in Figure 4c, untreated cells maintain a stable intracellular calcium concentration over an 80 s period (gray dotted line). In contrast, treatment with 50 μM **1P** caused a rapid increase in calcium concentration within 1 min, consistent with acute calcium influx triggered by plasma membrane disruption. Although disruption of the endoplasmic reticulum (ER) membrane can also cause an increase in intracellular Ca^2+^ levels, our additional results (Figures 4d, 5f–h, and 6) support plasma membrane disruption as the main source of the calcium concentration changes observed in Figure 4c.

**Figure 5:**
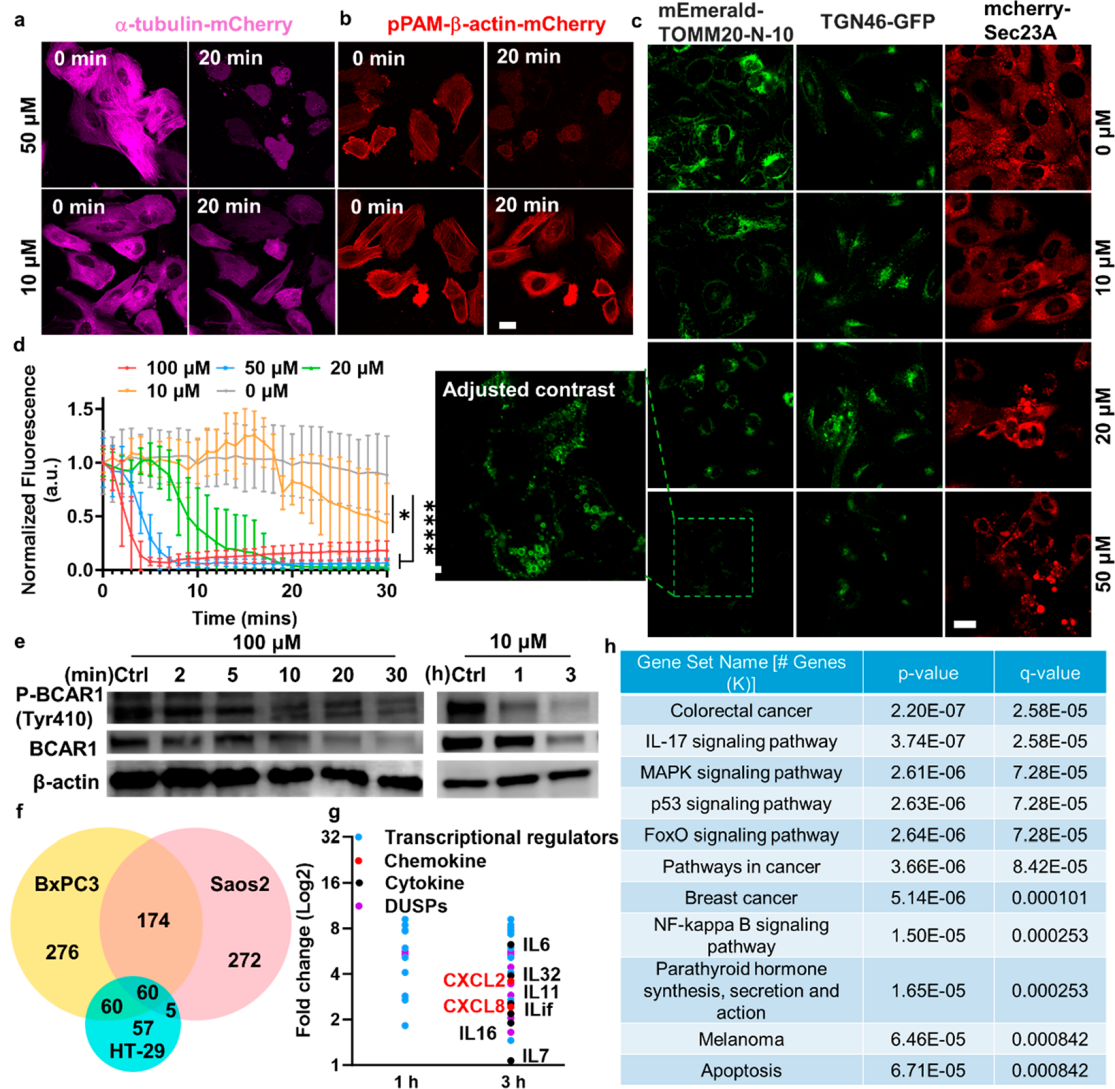
Membrane rupture triggers cytoskeletal collapse and organelle dysfunction. (a) Disruption of microtubules in Saos-2 cells treated with **1P** (10 or 50 μM). Scale bar, 20 μm. (b) Disruption of actin filaments in Saos-2 cells treated with **1P** (10 or 50 μM). Scale bar, 20 μm. (c) Morphological changes of mitochondria, Golgi apparatus, and endoplasmic reticulum (ER) in Saos-2 cells treated with **1P** (0, 10, 20, or 50 μM). Scale bar, 20 μm. Zoomed images show mitochondrial fragmentation in cells treated with 50 μM **1P** for 30 min (contrast adjusted). Scale bar, 10 μm. (d) Dose-dependent decrease in mitochondrial membrane potential of Saos-2 cells treated with **1P** (0–100 μM). (e) Western blot analysis of total and phosphorylated BCAR1 in Saos-2 cells treated with **1P** (10 or 100 μM) for the indicated times. (f) RNA-seq comparison of Saos-2 cells treated with **1P** (10 μM, 3 h) and published data sets from BxPC3 and HT-29 cells. Venn diagram shows overlapping transcriptional responses. (g) Upregulated cytokine and chemokine genes in Saos-2 cells treated with **1P** (10 μM). (h) Pathway enrichment analysis of differentially expressed genes, highlighting activation of MAPK and NF-κB signaling pathways.

**Figure 6:**
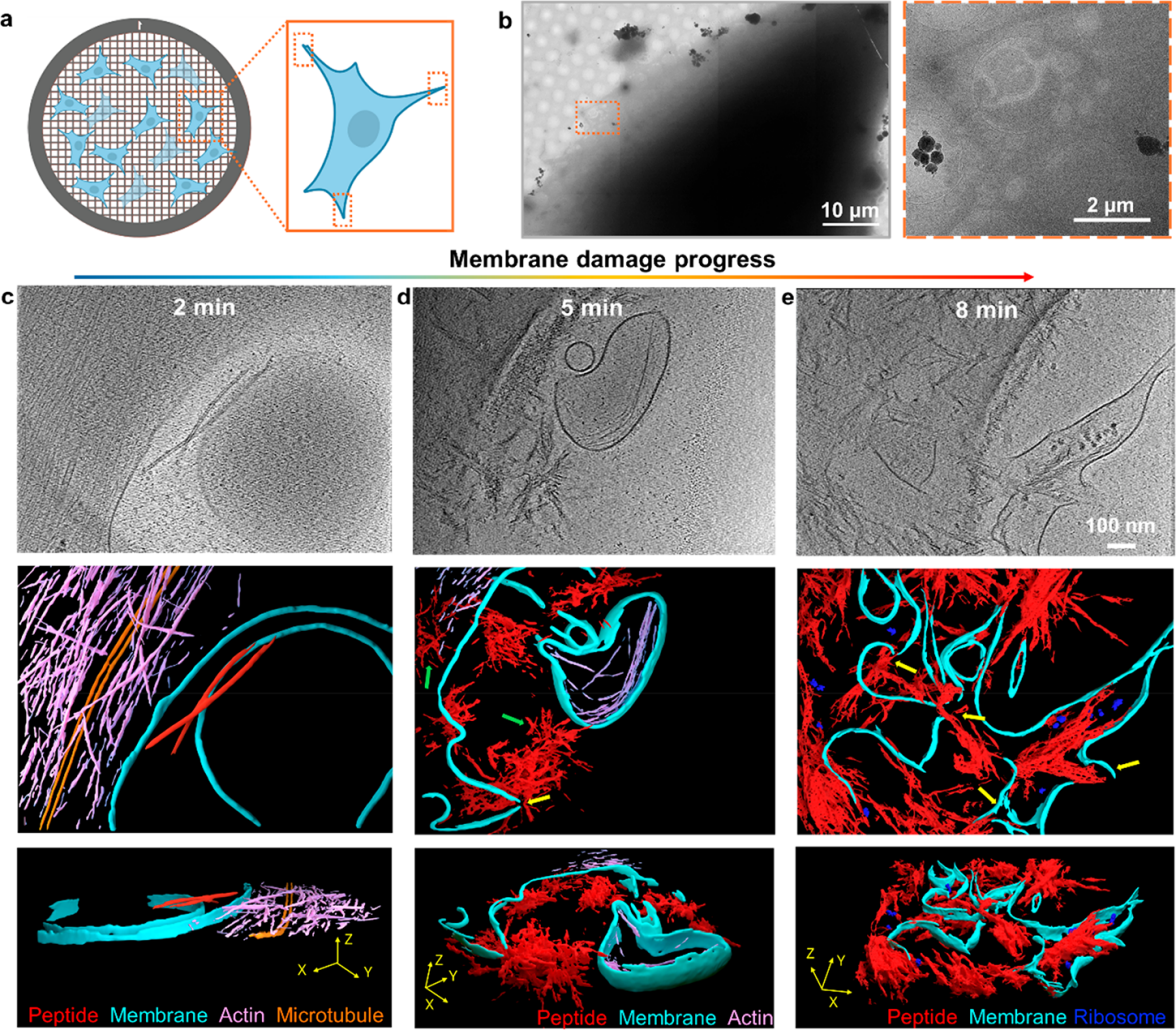
Cryo-ET and cryo-EM reveal the nanotube architecture underlying extrinsic lysis. (a) Schematic of Saos-2-cell sample on grid for cryo-ET imaging of the cell edge. (b) Cryo-EM images of the cell and zoom-in region of the cell edge. (c–e) Time-resolved cryo-tomograms and segmentation showing the interaction of **1P** (100 μM) with the plasma membrane of Saos-2 cells at (c) 2 min, (d) 5 min, and (e) 8 min. Red, peptide assemblies; light blue, plasma membrane; pink, actin filaments; orange, microtubules; blue, ribosomes.

To visualize morphological changes, we used a plasma membrane dye to monitor cell shape after **1P** exposure. Before treatment, cells were well spread with elongated morphology. Upon **1P** treatment, cells contracted, lost adhesion, and displayed increased height (Z dimension), indicative of structural collapse (Figure 4d). Residual membrane fragments delineated the original cell boundaries, suggesting membrane shedding during contraction (Video S1). These observations further support that **1P** compromises plasma membrane integrity.

Because calcium signaling activates membrane repair pathways involving the ESCRT machinery,^49^ we examined recruitment of CHMP4B, a key ESCRT component. We observed robust CHMP4B accumulation at the plasma membrane within 5 min of **1P** treatment (100 μM) (Figure 4e, yellow arrows, Figure S7), confirming that **1P** induces PMD sufficient to trigger the membrane repair response.

To determine whether **1P** induced membrane disruption engages canonical programmed cell death pathways, we pretreated Saos-2 cells with inhibitors of necroptosis (necrostain-1), pyroptosis (disulfiram), and ferroptosis (ferrostatin-1). None of these inhibitors rescued cell viability at either 10 or 100 μM **1P** (Figure 4f), indicating that **1P**-induced cytotoxicity is unlikely to rely on any single programmed death pathway.

Together, these data suggest that ALP-catalyzed peptide assembly forms supramolecular filaments that disrupt the plasma membrane, triggering calcium influx and transient repair attempts. When filament propagation exceeds PMR capacity, cells undergo irreversible cytolysis, consistent with a noncanonical, enzyme-triggered mechanism of extrinsic lytic cell death (Figure 4g).

### Membrane Rupture Cascades into Cytoskeletal Collapse and Organelle Failure

The plasma membrane and cytoskeleton act as an integrated mechanical system: when membrane integrity is lost, the underlying cytoskeletal network and organelle architecture rapidly deteriorate.^50^ We therefore investigated how **1P**-induced PMD influences cytoskeletal and organelle organization in Saos-2 cells. Live-cell imaging of cells expressing α-tubulin-mCherry or β-actin-pPAM-mCherry revealed rapid cytoskeletal disassembly following exposure to **1P**. Within minutes, 50 μM **1P** caused pronounced cell contraction, collapse of the microtubule network, and loss of α-tubulin fluorescence (Figure 5a, Video S1, and Supplementary Figure S8). At lower concentrations (≤20 μM), the effects were modest and reversible, indicating a concentration-dependent threshold for structural failure. F-actin architecture showed similar sensitivity: high **1P** doses disrupted filament organization and induced cortical condensation (Figure 5b, Video S2, and Supplementary Figure S9). Together, these observations demonstrate that iPF-induced PMD directly leads to cytoskeletal collapse.

Because the cytoskeleton maintains the spatial organization of intracellular organelles, its disruption may impair organelle structure and function. We next examined organelle integrity in Saos-2 cells expressing mEmerald-TOMM20-N-10 (mitochondria), TGN46-GFP (Golgi apparatus), and mCherry-Sec23A (endoplasmic reticulum (ER)). Increasing concentrations of **1P** led to a progressive decrease in mitochondrial fluorescence intensity and a morphological transition from tubular to rounded structures (Figure 5c, zoomed region), consistent with mitochondrial fragmentation or irreversible fission. To assess mitochondrial function, we measured the mitochondrial membrane potential. Exposure to 100 μM **1P** caused a rapid decline in potential, while lower concentrations produced slower depolarization (Figure 5d), confirming dose-dependent mitochondrial dysfunction. The Golgi apparatus also exhibited concentration-dependent morphological changes. As the **1P** levels increased, TGN46-GFP fluorescence initially intensified and formed numerous puncta, followed by a decline in both fluorescence and puncta numbers at higher doses (Figure 5c), suggesting Golgi fragmentation and collapse in this case. In cells expressing mCherry-Sec23A, **1P** (20–50 μM) induced the appearance of enlarged vesicular structures (Figure 5c). Because Sec23A is a component of the COPII complex mediates vesicle budding from the ribosome-free transitional ER,^51^ the emergence of these large vesicles suggests enhanced vesicle formation and disrupted trafficking. Collectively, these results indicate that **1P** treatment perturbs organelle organization, likely impairing vesicular transport and inducing cellular stress.

Because cytoskeletal anchoring links plasma membrane tension to signaling, we analyzed BCAR1 (p130Cas), a focal-adhesion adaptor mediating integrin-dependent mechanotransduction.^52^ Treatment with 100 μM **1P** caused a time-dependent decrease in both total and Tyr410-phosphorylated BCAR1 (Figure 5e). Prolonged exposure to 10 μM **1P** produced a similar reduction. The concurrent loss of BCAR1 and its phosphorylation suggests impaired focal adhesion signaling and weakened cytoskeletal tension, leading to reduced cell spreading and increased cell rounding (Figure 4d).

PMD is known to initiate a plasma membrane integrity (PMI) pathway,^53^ characterized by calcium-dependent recruitment of phosphorylated protein kinase C (pPKC-S660) and transcriptional activation of inflammatory mediators. Consistent with this model, **1P** treatment rapidly induced pPKC translocation to the plasma membrane (Figure 4e), confirming pathway engagement. RNA-sequencing of Saos-2 cells exposed to sublethal **1P** concentrations (10 μM, 3 h) revealed upregulation of PMI-associated cytokines and chemokines (CXCL2, CXCL8, IL6, IL7, LIF, and others) (Figure 5g), with strong overlap to previously reported PMI gene signatures^53^ (Figure 5f). Gene-set enrichment analysis further identified activation of the MAPK and NF-κB signaling pathways (Figure 5h), confirming that iPF-induced membrane rupture not only causes structural collapse but also triggers a coordinated transcriptional program associated with cellular stress and inflammation.

Collectively, these results indicate that enzyme-triggered supramolecular assemblies convert a biochemical reaction into a mechanical failure event that propagates across cellular scales—from plasma membrane rupture to cytoskeletal collapse, organelle dysfunction, and activation of stress signaling. This cascade defines the cellular execution phase of extrinsic lytic death.

### Cryo-ET Reveal Nanotube Architecture Underlying Extrinsic Lysis

To elucidate how ALP-triggered peptide assemblies interact with the plasma membrane at nanometer resolution, we used time-resolved cryo-ET to reveal the spatiotemporal progression of the iPFs-membrane interactions *in situ* responsible for extrinsic lytic death. To visualize how iPFs assemble at cell periphery and interact with intracellular components at nanometer resolution, we cultured Saos-2 cells on cryo-EM grids, then plunge-froze them and used cryogenic focused ion beam (cryo-FIB) milling to generate lamellae thinner than 200 nm for cryo-ET imaging (Figures 6a and S10a). The cell periphery is naturally thin, enabling high-contrast tomography imaging without sample thinning. (Figure 6a). Cell body, however, requires cryo-FIB milling to generate lamella thinner than 200 nm for imaging (Figure S10b). Cryo-EM images revealed membrane invagination and vesicle-like structures (Figure 6b), providing suitable regions for 3D reconstruction of membrane architecture. The time-resolved cryo-ET tomograms provide an internal, time-dependent control, revealing the emergence of exogenous needle-like, entangled assemblies that increase over time and progressively disrupt the plasma membrane. This time-dependent behavior allows us to distinguish and segment these assemblies from endogenous cellular components.

Given the rapid onset of peptide-membrane interactions, we performed time-resolved cryo-ET to capture early structural intermediates at the cell periphery. We selected the time points of 2, 5, and 8 min based on live-cell imaging, which showed peptide entry, membrane blebbing, and cell lysis happened sequentially (Figure 6c, d, and e).

At 2 min, elongated peptide filaments closely associated with plasma membrane (Figure 6c and Video S3). At this time, the membrane and vesicles remain smooth, and intracellular actin and microtubules remain intact, indicating minimal mechanical stress. Moreover, the filament diameter (∼11 nm) matched that determined by in vitro cryo-EM analysis (Figure 2i), consistent with phosphorylated iPFs and excluding their misassignment to endogenous cytoskeletal filaments.

At 5 min, these filaments transformed into nanoclusters (Figure 6d and Video S4), consistent with the ALP-mediated dephosphorylation at membrane surface. We observed actin-encapsulated extracellular vesicles, suggesting that Saos-2 cells expelled damaged membrane fragments as a self-protective response, consistent with the observations in Figure 5d. Moreover, local membrane discontinuities (yellow arrow, Figure 6d) and peptide assemblies (green arrow, Figure 6d) on both sides of the bilayer indicated the onset of leakage and transmembrane penetration.

At 8 min, the plasma membrane displayed extensive rupture (Figure 6e and Video S5). At this time, peptide assemblies spanned across the disrupted membrane (indicated by arrow), making the intracellular and extracellular space indistinguishable, indicating complete loss of membrane integrity (Figure 6e).

Following PMD, cryo-ET of the cytoplasmic lamellae revealed dispersed nanoclusters throughout the intracellular space (Figure S10). Segmentation and 3D reconstruction of the tomograms showed dense peptide assemblies localized near mitochondria and the endoplasmic reticulum (ER) (Figure S10), indicating possible organelle stress and dysfunction at later stage after the membrane rupture. The mitochondria appeared rounded and fragmented, consistent with mitochondrial fission and depolarization seen in fluorescence microscopy (Figure 5c). The morphology of cytoplasmic assemblies mirrored those at cell edge, supporting a sequential mechanism: ALP catalyzes peptide assembly at the membrane, filaments grow and penetrate the membrane, followed by intracellular migration of assemblies that accumulate at ER and mitochondria, leading to organelle stress and dysfunction.

The cryo-ET, together with cryo-EM data and the imaging and biochemical assays, establish a unified, cross-scale mechanism (Figure 1b): (i) ALP dephosphorylates **1P** at the plasma membrane. (ii) The dephosphorylated peptide self-assembles into rigid, dimer-based filaments. (iii) The filaments grow along and across the membrane, deforming and rupturing it. (iv) Cells undergo cytoskeletal collapse, organelle failure, and irreversible lysis. This cryo-structural mechanism shows how enzymatic catalysis drives supramolecular order that physically destroys cell membranes. Unlike pore-forming proteins or small-molecule toxins, this process transforms a tumor-promoting enzyme into an executor of membrane rupture. ALP-triggered self-assembly therefore provides a generalizable strategy to exploit enzyme-regulated supramolecular chemistry for targeted cell elimination.

## Conclusion

In summary, this study used time-dependent cryo-ET to establishes a molecular mechanism of extrinsic lytic cell death driven by enzyme-instructed peptide self-assembly. Tumor-associated ALP catalyzes the dephosphorylation of a designed phospho-biphenyl peptide (**1P**), triggering the rapid formation of iPFs on the cancer cell surfaces. These extracellular filaments breach the plasma membrane, overwhelm repair responses, and initiate a cascade of cytoskeletal collapse, organelle dysfunction, and transcriptional stress signaling that culminates in irreversible cytolysis. By integrating cryo-EM and cryo-ET, we directly visualize this process across molecular and cellular scales: while cryo-EM resolves the dimeric nanotube architecture at 2.5–2.9 Å resolution, cryo-ET captures iPFs penetrating the plasma membrane in live cells. Together, these analyses reveal how enzyme-catalyzed supramolecular order is transduced into mechanical membrane rupture. These molecular insights are significant for further developing or optimizing the therapeutics that are based on enzymatic self-assemblies^54,26,55–58,34,59–66^ or peptide assemblies.^67–71^

More broadly, this work confirms the functional potential of EISA for targeting membranes as a way to kill cancer cells via “sabotage”.^72^ By coupling enzymatic catalysis to supramolecular mechanics, we demonstrate how molecular design can program structural order to dictate cell fate. This strategy establishes a supramolecular chemical biology framework for exploiting pathological enzyme activity to eliminate resistant cancer cells. Designing rigid peptide assemblies that target other membrane-associated enzymes could generalize this approach, extending structure-guided, enzyme-regulated self-assembly into a new modality of selective anticancer intervention.

## Supplementary Material





## Notes

Detailed experimental procedures and materials; LC/MS results of individual compounds; additional CLMS images (PDF)
Videos S1–S5 (ZIP)

## Abbreviations

ACP: acid phosphatase
ALP: alkaline phosphatase
ATP: adenesine triphosphate
BNPP: bis­(4-nitrophenyl) phosphate
CAC: aggregation concentration
CHMP4B: charged multivesicular body protein 4B
cryo-EM: cryogenic electron microscopy
CXCL2: C-X-C motif chemokine ligand 2
cryo-ET: cryogenic electron tomography
cryo-FIB: cryogenic focused ion beam
CTL: cytotoxic T lymphocyte
CXCL8: interleukin 8
DQB: 2,5-dimethoxy-N-(quinolin-3-yl)­benzamide
EISA: enzyme-instructed peptide self-assembly
ER: endoplasmic reticulum
ESCRT: endosomal sorting complex required for transport
ff: di-d-phenylalanine
IL6: interleukin 6
IL7: interleukin 7
iPFs: in situ peptide filaments
GSDMD: gasdermin D
LDH: lactate dehydrogenase
LIF: leukemia inhibitory factor
MLKL: mixed lineage kinase domain like pseudokinase
pBP: phospho-biphenyl
PKC: protein kinase C
PMD: plasma membrane damage
PMI: plasma membrane integrity
PMR: plasma membrane repair
RIPK3: receptor-interacting protein kinase 3
TEM: transmission electron microscopy.
