# Supplementary material for: Cryo-Structural Insights into Enzymatic Peptide Self-Assembly Driving Extrinsic Lytic Cell Death
Source: J Am Chem Soc. 2026 Mar 24;148(13):14117–28. doi: 10.1021/jacs.5c23283 (PMC13067268; doi:10.1021/jacs.5c23283)
Supplement: Supplementary file 1 [file ja5c23283_si_001.pdf]

Supplementary Information for

Cryo-Structural Insights into Enzymatic Peptide Self-assembly Driving Extrinsic Lytic Cell Death

Meihui Yi[1], Jiaqi Guo[2,3], Ayisha Zia[4], Wangbiao Guo[2,3], Shoichi Tachiyama[2,3], Gabriel Ashton-Rickardt[1], Weiyi Tan[1], Yuchen Qiao[1], Yinan Gong[6], Edward H. Egelman[5], Jun Liu[2,3], Fengbin Wang[4,5,*], and Bing Xu[1,*]

1. Department of Chemistry, Brandeis University, Waltham, MA 02454, USA.
2. Microbial Sciences Institute, Yale University, West Haven, CT 06516, USA
3. Department of Microbial Pathogenesis, Yale School of Medicine, New Haven, CT 06536, USA
4. Department of Biochemistry and Molecular Genetics, University of Alabama at Birmingham, Birmingham, AL, 35233, USA.
5. Department of Biochemistry and Molecular Genetics, University of Virginia, Charlottesville, Virginia, 22908, United States
6. Department of Immunology, University of Pittsburgh and UPMC Hillman Cancer Center, Pittsburgh, PA 15232, USA

Contents

Experiment materials and instruments 5

Materials 5

Instruments 5

Chemical synthesis 6

Synthesis of phosphobiphenyl carboxylic acid 6

Synthesis of peptide derivatives (SPPS) 6

Attaching NBD to the side chain of pBPffk 7

Methylation of short peptides 8

Critical aggregation concentration (CAC) measurement 8

Gelation experiment 8

Negative staining TEM sample preparation 9

Cryo-EM microscope and image processing 9

Model building of nanotubes 10

Cell culture 10

MTT assay 11

LDH assay 12

Confocal laser scanning microscopy (CLSM) 12

CLSM imaging of cellular uptake of 2P 13

CLSM imaging of membrane disruption 13

CLSM imaging of cytoskeleton disruption 13

Immunocytochemistry (ICC) 14

Cryo-ET 14

Supplementary Figures 16

Figure S1. GI_{50} and GI_{90} values of 1P against Saos2, SJSA1, HepG2, and HS5 cells after 24 h. 16

Figure S2. The cell viability of Saos2, SJSA1, HS5 and HepG2 treated with 1P for 24 h, 48 h, and 72 h. 17

Figure S4. a. The illustration of adding extra alkaline phosphatase on the cell surface of HS-5. b. The cell viability of HS-5 or HS-5 with extra AP treated with 1P for 24 h. 17

Figure S5. a. The cell viability of HepG2 cells treated with BNPP, an inhibitor to carboxylic esterase for 24 h. B. The cell viability of HepG2 cells treated with 1P, and 1P with coincubation of BNPP for 24 h. 19

Figure S6. The cellular uptake of 2P (20 μM) by Saos2 with or without the coincubation of DQB (20 μM). 20

Figure S7. The plasma membrane translocation of CHMP4B and pPKC(S660) of Saos2 with the treatment of 1P (100 μM) at different time points. The control group without the treatment of primary antibody. 21

Figure S8. The disruption of tubulin structures of α-tubulin-mCherry transfected Saos2 cells with the treatment of 1P (0, 10, 20, 50, and 100 μM) over 20 minutes. The scale bar is 20 μm. 22

Figure S9. The disruption of F-actin structures of pPAM-β-actin-mCherry transfected Saos2 cells with the treatment of 1P (0, 10, 20, 50, and 100 μM) over 20 minutes. The scale bar is 20 μm. 23

Figure S10. a. SEM image of several Saos2 cells, incubated by 1P (200 μM), on an EM grid. Scale bar 5 μm. b. A SEM image of the Saos2 cell after cryo-FIB milling. c. A cryo-ET image shows an extensive bundle of nanofibers inside the Saos2 cell. Scale bar 100 nm. 24

Figure S11. The LC spectrum of 1P (inset) and its corresponding mass spectrum. 24

Figure S12. The LC spectrum of 2P (inset) and its corresponding mass spectrum. 25

Table S1. Cryo-EM and refinement statistics 26

Video S1. The morphological change of tubulin of Saos2 treated with 50 μM 1P for 30 minutes. 27

Video S2. The morphological change of actin of Saos2 treated with 50 μM 1P for 30 minutes. 27

Video S3. Time-resolved cryo-tomograms and segmentation showing the interaction of 1P (100 μM) with the plasma membrane of Saos2 at 2 minutes. 27

Video S4. Time-resolved cryo-tomograms and segmentation showing the interaction of 1P (100 μM) with the plasma membrane of Saos2 at 5 minutes.......**27**

Video S5. Time-resolved cryo-tomograms and segmentation showing the interaction of 1P (100 μM) with the plasma membrane of Saos2 at 8 minutes.......**27**

Experiment materials and instruments

Materials

2-Cl-trityl chloride resin (1.0-1.2 mmol/g) and HBTU were obtained from GL Biochem (Shanghai, China). 4'-hydroxy-[1,1'-biphenyl]-4-carboxylic acid was obtained from 1PlusChem. Benzene and phosphorus pentachloride was purchased from Sigma-Aldrich. 4-Chloro-7-nitrobenzofurazan was purchased from Alfa Aesar. Other chemical reagents and solvents were obtained from Fisher Scientific. Alkaline phosphatase was purchased from Biomatik (Cat. No. A1130, alkaline phosphatase [ALP], >1300U/mg, in 50% glycerol.). Minimum Essential Media (MEM), Dulbecco's Modified Eagle Medium (DMEM), McCoy's 5A Medium, and RPMI-1640 Medium were purchased from ATCC. Fetal bovine serum (FBS) and Penicillin-Streptomycin from Gibco by Life Technologies. All chemical reagents and solvents were used as received from commercial sources without further purification.

Instruments

All precursors and compounds were purified by a reverse phase HPLC (Agilent 1100 Series) equipped with an XTerra C18 RP column, and HPLC grade acetonitrile (0.1% TFA) and HPLC grade water (0.1% TFA) were used as the eluents. The LC-MS spectra were obtained with a Waters Acquity Ultra Performance LC with Waters MICROMASS detector. TEM images were taken on a Morgagni 268 transmission electron microscope. The absorbance of each well at 595 nm was measured by a DTX880 Multimode Detector. The confocal images are obtained by using Zeiss LSM 880 confocal microscopy and Nikon AX-R CLSM at the lens of 63× with oil.

Chemical synthesis

Synthesis of phosphobiphenyl carboxylic acid

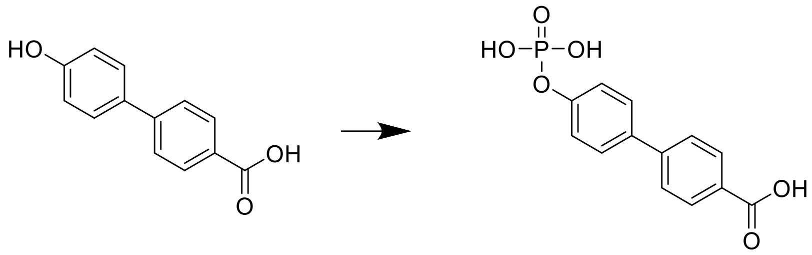

Scheme S1. The synthesis of phosphobiphenyl group.

4’-hydroxy-[1,1’-biphenyl]-4-carboxylic acid (1 eq.) and PCl_5 (1 eq.) were stirred at 60 ℃ for 90 min. The ice-cooled reaction mixture was dissolved in 10 mL of acetone and 10 mL of benzene, and 1.4 mL (3 eq.) of distilled water was added dropwise. After stirring at 0 ℃ for 30 min, 20 mL benzene was added. The reaction mixture was stirred at room temperature for 12 h. The precipitate was filtered off, washed with 20 mL benzene, and dried in high vacuum.

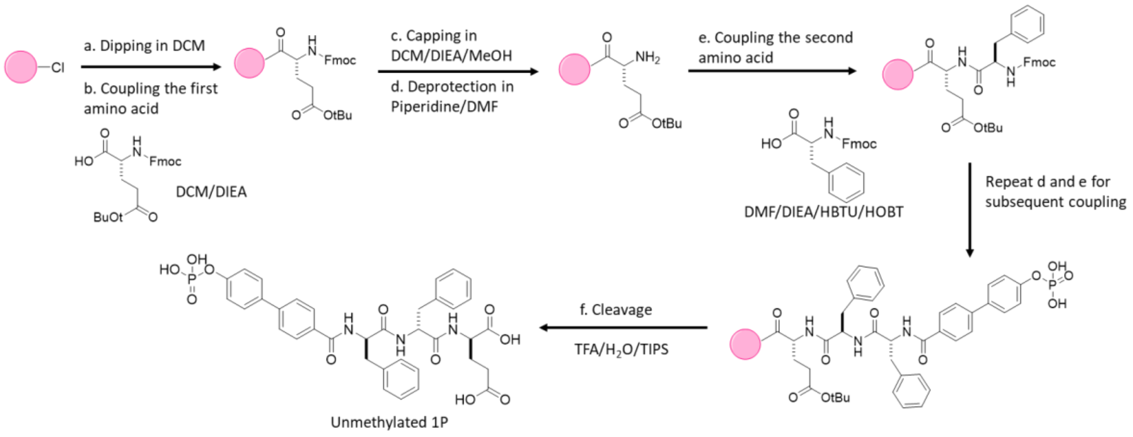

Scheme S2. The synthesis of peptides derivatives without methylation.

Synthesis of peptide derivatives (SPPS)

After synthesis of phosphobiphenyl group, we first synthesized peptides derivatives without methylation using standard Fmoc solid phase peptide synthesis. This involves

utilizing 2-chlorotrityl chloride resin and the corresponding Fmoc-protected amino acids with side chains properly protected.

Here is the breakdown of the synthesis steps:

a. The 2-chlorotrityl chloride resin (0.5 g) was weighed and immersed in methylene chloride (DCM) for 15 min.

b. The amino acids were weighed according to 1.2 mmol/g of resin. The amino acid (2 equivalent) was dissolved in DCM with the addition of 5 equivalent of N, N-Diisopropylethylamine (DIEA), then the resin was mixed with the solution well on a rocker for 1 h. DCM was used for washing the resin.

c. The capping solution (DCM: MeOH: DIEA= 17: 2: 1) was added and allowed to react for 15 min. DCM was used for initial washing, followed using dimethylformamide (DMF) for further washing.

d. To remove the Fmoc group, 20% piperidine in DMF was added and left to react for 30 min. DMF was used for washing.

e. Amino acid (2 equivalent), HBTU (2 equivalent), and DIEA (5 equivalent) in DMF were loaded and reacted for 40 min. DMF was used for washing.

f. DCM was used to wash the remaining DMF. The peptide cleavage was performed by using 95% TFA, 2.5% triisopropyl silane (TIPS), and 2.5% H_2O for 30 min.

Attaching NBD to the side chain of pBPffk

To a solution of pBP-ffk (1 eq.) and Na_2CO_3 (2 eq.) in H_2O was added NBD-Cl (1 eq.) dissolved in methanol. The ratio of H_2O to methanol is 1:3. The mixture was stirred at 60 °C for 2 h, and then cooled to room temperature, neutralized with 10% HCl (aq). The solvent was air dried, and the crude product was purified by HPLC.

Methylation of short peptides

pBP-ff (1 equivalent) was dissolved in DCM with stirring and then bromotrimethylsaline (15 equivalent) was added. The reaction mixture was stirred at room temperature overnight. After air dried, methanol was added, and the reaction was stirred at room temperature for 1 day. Then the product was purified with HPLC.

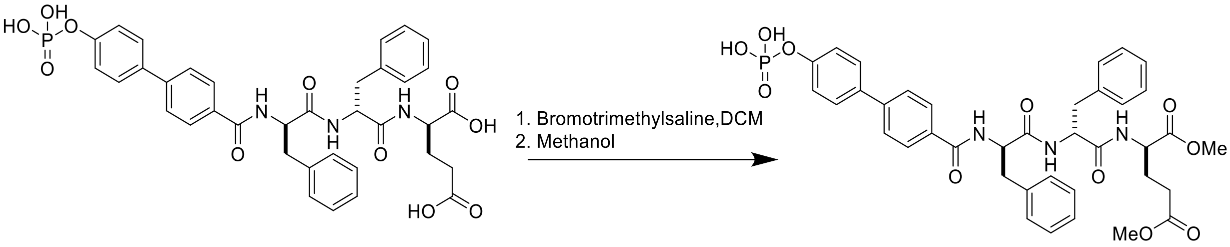

Scheme S3. The methylation of pBP-ffe. Same procedure was used to methylate other derivatives.

Critical aggregation concentration (CAC) measurement

A series of precursor (1P) solutions from the concentration of 4 mM to 0.2 μM was prepared in pH 7.4 PBS buffer, with or without the treatment of 1 U/mL ALP. After incubating with Rhodamine 6G (5 μM), the λ_{max} was determined by measuring the absorbance from 520 to 540 nm using a Biotek Synergy 4 hybrid multi-mode microplate reader.

Gelation experiment

Gelation experiments were carried out in 1.5 mL glass vials. 1 mg of 1P was first dissolved in 100 μL of 1x PBS buffer. The pH of the solution was carefully adjusted to 7.4 with 1 M NaOH (aq). Then extra PBS buffer was added to make the solutions with the volume of 200 μL and final concentration of 0.5 wt%. 1 μL of 0.2 U μL^{-1} ALP was

added to make the final concentration of 1 U mL^{-1}. After incubation at room temperature for 24 h, the hydrogel was formed.

Negative staining TEM sample preparation

a. Place sample solution on the grid (5 μL, sufficient to cover the grid surface).

b. Rinsing: ~10 sec later, place a large drop of the ddH_2O on parafilm and let the grid touch the water drop, with the sample-loaded surface facing the parafilm. Tilt the grid and gently absorb water from the edge of the grid using a filter paper sliver. (3 times)

c. Staining (immediately after rinsing): place a large drop of the UA (uranyl acetate) stain solution on parafilm and let the grid touch the stain solution drop, with the sample-loaded surface facing the parafilm. Tilt the grid and gently absorb the stain solution from the edge of the grid using a filter paper sliver.

d. Allow the grid to dry in air and examine the grid as soon as possible.

Cryo-EM microscope and image processing

The propeptide nanotubes sample was applied to glow-discharged lacey carbon grids and vitrified using a Leica plunge freezer (Leica). Grids were imaged on a 300 keV Titan Krios (Thermo Fisher) with a K3 camera (Gatan). Micrographs were collected under electron counting mode, using a defocus range of 1–2 μm with ~50 electrons/$Å^2$ distributed into 40 fractions. Motion correction and CTF estimation were done in cryoSPARC[1-3]. Particles were auto-picked by "Filament Tracer" with a shift of ~12 pixels. Non-peptide junk particles were removed by multiple rounds of reference-free 2D classifications. Particles were kept if they have clear 2D average patterns. Nanotubes with different diameters were sorted based on 1D projections of their 2D class averages. Classes sharing the same peak-to-peak distance in their 1D projections were then grouped into a single subgroup. This process was done

iteratively to get homogenous subgroups of particles. This approach is used because out-of-plane tilt does not affect the peak-to-peak distance in these 1D projections. For each class of nanotubes, the possible helical symmetries were calculated from an averaged power spectrum of the raw particles. All possible symmetries were then tested by trial and error in cryoSPARC until recognized peptide features, such as density of side chains, were observed[4]. The resolution of each reconstruction was estimated by both Map:Map FSC and Model:Map FSC. The final volumes were then sharpened with a negative B-factor automatically estimated in cryoSPARC, and the statistics are listed in Table S1.

Model building of nanotubes

Since those nanotubes are held together by β-sheets and π-π interactions, the hand of the helical map cannot be determined directly from the cryo-EM volume. This is unlike volumes that contain an α-helix, in which the hand is obvious when the resolution is 4.5 Å or better. In the published cross-β structures, the parallel β-sheets made of L-peptide typically have a left-handed twist. However, this observation may not be deducible to short peptide containing non-standard residues. Therefore, we did model building in both hands of maps. First the model was manually adjusting in Coot[5] and then real-space refined in PHENIX[6]. It turned out all models fits in right-handed protofilaments (18-, 17-, 16-, 15-, respectively) slightly better than the reserve hand, with slightly higher RSCC and better Clashscore. This is consistent with previous structures as the peptide used in this study are D-peptides. The resolutions for all four classes and their refinement statistics are shown in Table S1.

Cell culture

Saos2, SJSA1, HS5 and HepG2 cell lines were purchased from American Type

Culture Collection (ATCC, USA). Saos2 cells were cultured in McCoy's 5A Medium (Gibco, Life Technologies) supplemented with 15% (v/v) fetal bovine serum (FBS) (Gibco, Life Technologies), 100 U/mL penicillin and 100 μg/mL streptomycin (Gibco, Life Technologies); SJSA1 cell were culture in RPMI1640 (ATCC, USA) Medium supplemented with 10% (v/v) FBS, 100 U/mL penicillin and 100 μg/mL streptomycin; HepG2 cells were cultured in Minimal Essential Medium (MEM) (Gibco, Life Technologies) supplemented with 10% (v/v) FBS, 100 U/mL penicillin and 100 μg/mL streptomycin. HS5 cells were cultured in Dulbecco's Modified Eagle Medium (DMEM) supplemented with 10% (v/v) FBS, 100 U/mL penicillin and 100 μg/mL streptomycin. All the cells were maintained at 37 °C in a humidified atmosphere of 5% CO_2.

MTT assay

Cells were seeded in 96-well plates at 1×10^4 cells/well for 24 hours to allow attachment. After removing the culture medium, fresh culture medium containing different concentration of the precursors were added. After 24/48/72 hours, 10 μL MTT (ACROS Organics) solution (5 mg/mL) was added to each well to incubate at 37 °C for 4 h. 100 μL of SDS-HCl solution was then added to stop the reduction reaction and dissolve the formazan. The absorbance of each well at 595 nm was measured by a DTX880 Multimode Detector. The results were calculated as cell viability percentage relative to untreated cells. The MTT assay was performed in triplet (n = 3) and the average value of the three measurements was taken.

For the ALPL inhibitor rescue experiment, after cell attachment, the cells were pretreated with ALPL inhibitors for 30 minutes, followed by co-culture with a mixture of **1P** and ALPL inhibitors. After 2 hours, the same procedures were performed to determine cell viability as a percentage relative to untreated cells.

For the cell death pathway inhibitor rescue experiment, after cell attachment, the cells

were pretreated with death pathway inhibitors for 2 hours, then co-cultured with a mixture of **1P** and different inhibitors. After 2 hours, cell viability was assessed using the same procedures, with results expressed as a percentage relative to untreated cells.

LDH assay

LDH release (%) was measured using Pierce™ LDH Cytotoxicity Assay Kit purchased from Thermo Scientific™, using the protocols as following:

1. Seed Saos2 cells at 1×10^5 cells/well in triplicate wells in a 96-well plate for 24 hours to allow attachment.
2. Remove culture medium and incubate the cells with the 1P at different concentrations.
3. After incubation with precursors at different time points, transfer 50 μL of each sample medium to a 96-well plate.
4. Add 50 μL Reaction Mixture to each sample well and mix gently.
5. After incubating the plate at room temperature for 30 minutes protected from light, add 50 μL of Stop Solution to each sample well and mix by gentle tapping.
6. Measure the absorbance at 490 and 680 nm using a Biotek Synergy 4 hybrid multi-mode microplate reader. Before calculation of LDH release, subtract the 680 nm absorbance value (background) from the 490 nm absorbance [(LDH at 490 nm) - (LDH at 680 nm)]. The LDH release (%) was calculated as percentage relative to maximum LDH activity controls.

Confocal laser scanning microscopy (CLSM)

Confocal dish (35 mm dish with a 20 mm #1.5 glass bottom well, Cellvis) was used for preparing CLSM samples. All cells were directly seeded on the confocal dish without additional coating.

CLSM imaging of cellular uptake of 2P

Saos2 were seeded on a confocal dish at 1.0×10^5 cells/dish for 24 h. After aspirating the culture medium, cells were pretreated with 20 μM DQB for 30 minutes. Then, the samples were brought to Nikon AX-R CLSM. Following removal of the old medium, fresh culture medium containing 20 μM 2P either alone or in combination with 20 μM DQB was added. The fluorescence images with channels (488 nm) of the sample were taken every minute for 30 minutes and saved for further analysis.

CLSM imaging of membrane disruption

Saos2 were seeded on a confocal dish at 1.0×10^5 cells/dish for 24 h. Then, the samples were brought to ZEISS LSM 880 confocal laser scanning microscope. Following the removal of culture medium, fresh culture medium containing **1P** of different concentrations (10 μM or 50 μM) together with Alexa-fluo-647-hydrazide (2 μM) were added. The fluorescence images with channels (633 nm) of the sample were taken every minute for 30 minutes and saved for further analysis.

CLSM imaging of cytoskeleton disruption

Saos2 expressing α-tubulin-mCherry or β-actin-pPAM-mCherry were seeded on a confocal dish at 1.0×10^5 cells/dish for 24 h. Then, the samples were brought to Nikon AX-R CLSM. Following the removal of culture medium, fresh culture medium containing **1P** of different concentrations (10 μM or 50 μM) were added. Fluorescence images (561 nm channel) of Saos-2 cells expressing α-tubulin-mCherry were acquired every minute for 30 minutes and saved for subsequent analysis. For Saos-2 cells expressing β-actin-pPAM-mCherry, the cells were first activated with a 405-nm laser, after which fluorescence images in the 561-nm channel were collected every minute

for 30 minutes and saved for further analysis.

Immunocytochemistry (ICC)

Saos2 were seeded on a corning 96-well plate at 1.0×10^5 cells/mL for 24 h. After removing the culture medium, fresh medium containing 100 μM **1P** was added for 2, 5, 10, 20, or 30 minutes. Following **1P** removal, cells were fixed with 4% parformaldehyde (PFA) for 10 minutes. After three washes with PBS, cells were permeabilized with 0.1% Triton X-100 in PBS for 10 minutes, then washed three more times with PBS. After blocking with 2% BSA in PBS for 30 minutes, the primary antibody (1:200 dilution for CHMP4B and 1:500 dilution for pPKC(S660)) in 2% BSA was added and incubated overnight in 4 ℃. After five washes with PBS, Alexa Fluor 647-conjudated secondary antibody (1:1000 dilution) in 2% BSA was added for 1h at room temperature. After five washes with PBS, the sample was taken to Nikon AX confocal system for imaging and saved for further analysis.

Cryo-ET

Saos-2 cells were seeded onto glow-discharged gold EM grids and allowed to attach for 24 h before treating with 100 μM peptide for defined time intervals (2, 5, or 8 min, or 2 h). After incubation, excess medium was removed, grids were briefly washed with culture medium containing 10% glycerol and immediately plunge-frozen in liquid ethane. Grids of the 2 h treatment sample were clipped with cryo-FIB AutoGrids for lamella preparation, whereas grids from the early time points were clipped and used directly for cryo-ET.

Cryo-FIB was performed on a Aquilos 2 cryo-FIB/SEM (Thermo Fisher Scientific). Low-magnification SEM montages were acquired in MAPS to identify milling sites. Grids were sputter-coated with metallic platinum (30 mA, 15 s), followed by a 25 s

deposition of organometallic platinum and an additional 15 s metallic platinum layer to minimize beam-induced charging. Lamellae were produced by sequential milling at 0.3 nA to ~1 μm, 50 pA to ~500 nm, and final polishing at 30 pA, resulting in lamellae <200 nm thick. SEM inspection confirmed preservation of cellular components, and lamellae were stored in liquid nitrogen until imaging.

Tilt series of lamella were collected on a Titan Krios (Thermo Fisher Scientific) equipped with a Gatan K3 direct electron detector. Cell peripheries were imaged on a Glacios microscope with the same detector. Data were acquired in SerialEM using FASTtomo scripts[7] with a dose-symmetric scheme (±48° range, 3° increments; total dose ~70 e^{-} $Å^{-2}$) at a pixel size of 2.148 Å (Krios) or 2.346 Å (Glacios) and a defocus of ~–6 μm. Movie frames (~33 per tilt) were aligned in MotionCor2[2] and assembled into drift-corrected stacks in IMOD[8]. Fiducial-less patch tracking alignment and tomogram reconstruction were performed in IMOD[9]. Segmentation was carried out in Dragonfly, and 3D visualization and rendering were performed in Dragonfly and Blender.

Supplementary Figures

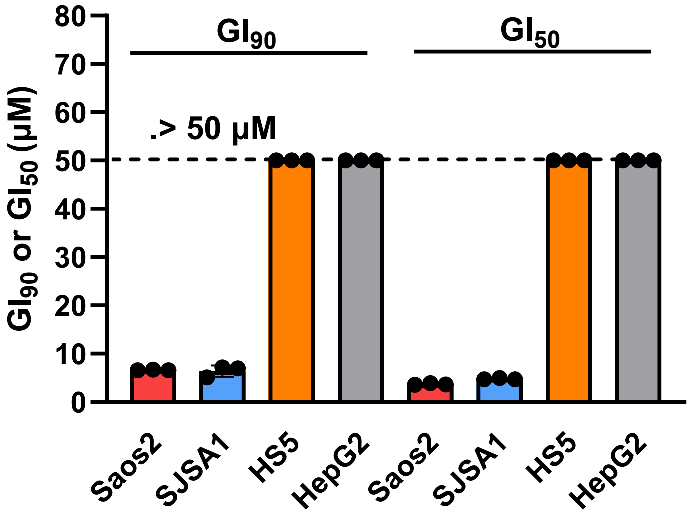

Figure S1. GI_{50} and GI_{90} values of 1P against Saos2, SJSA1, HepG2, and HS5 cells after 24 h.

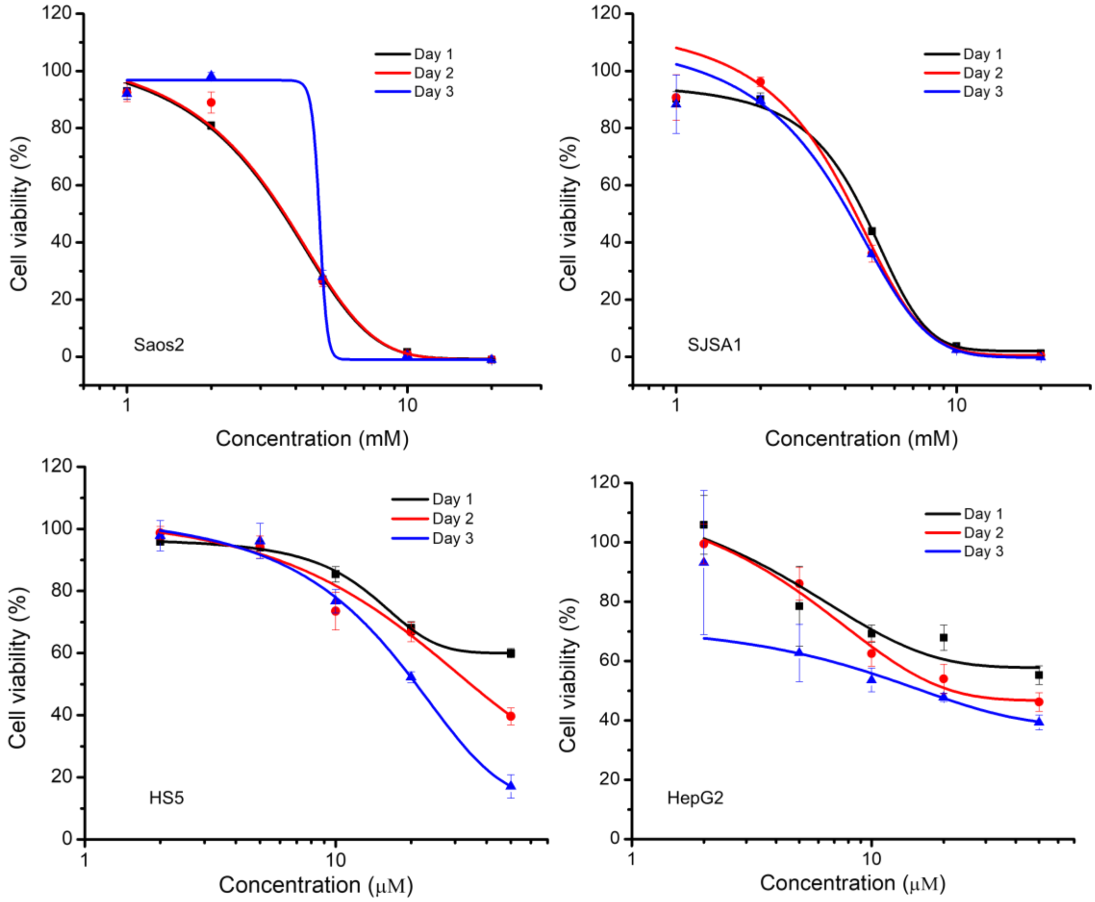

Figure S2. The cell viability of Saos2, SJSA1, HS5 and HepG2 treated with 1P for 24 h, 48 h, and 72 h.

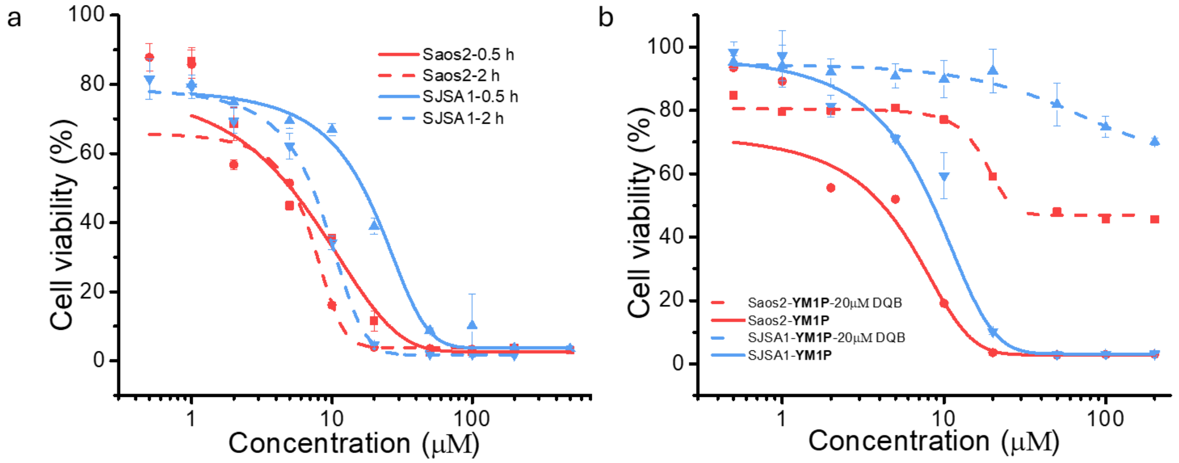

Figure S3. a. The cell viability of Saos2 and SJSA1 treated with 1P within 0.5 h or 2 h. b. The cell viability of Saos2 and SJSA1 treated with 1P or the coincubation of 1P with DQB for 2 h.

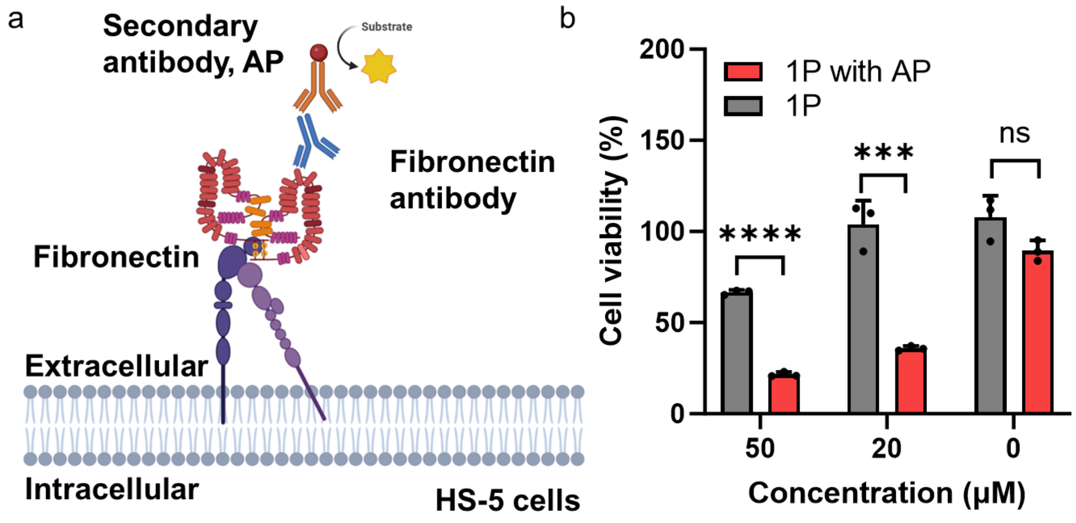

Figure S4. a. The illustration of adding extra alkaline phosphatase on the cell surface of HS-5. b. The cell viability of HS-5 or HS-5 with extra AP treated with 1P for 24 h.

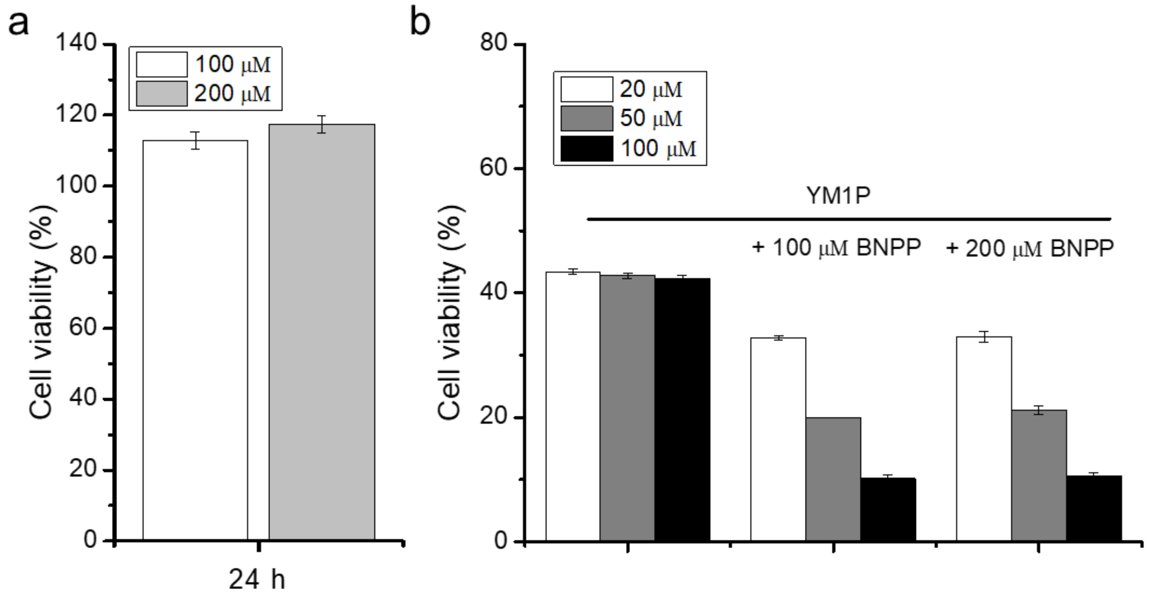
a
b
Cell viability (%)
100 μM
200 μM
24 h
20 μM
50 μM
100 μM
YM1P
+ 100 μM BNPP
+ 200 μM BNPP

Figure S5. a. The cell viability of HepG2 cells treated with BNPP, an inhibitor to carboxylic esterase for 24 h. B. The cell viability of HepG2 cells treated with 1P, and 1P with coincubation of BNPP for 24 h.

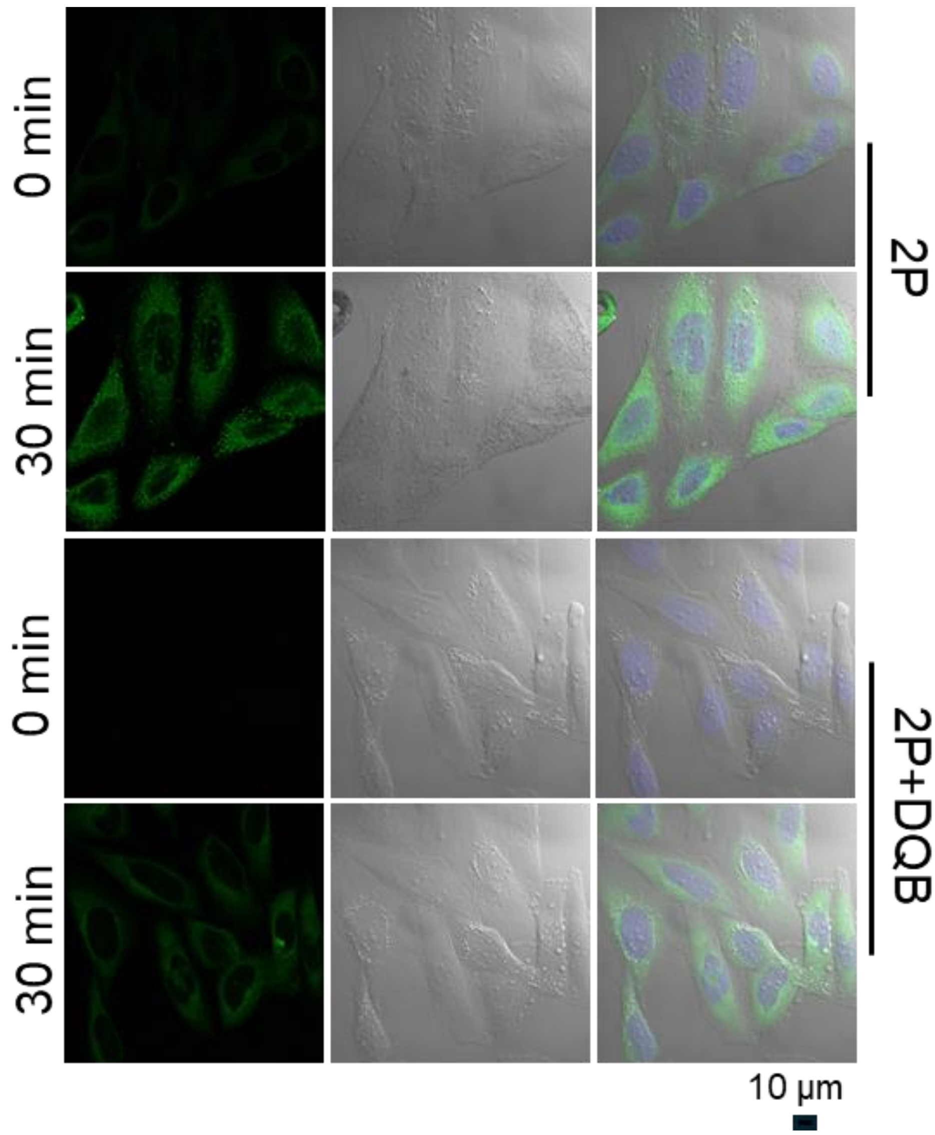

Figure S6. The cellular uptake of 2P (20 μM) by Saos2 with or without the coincubation of DQB (20 μM).

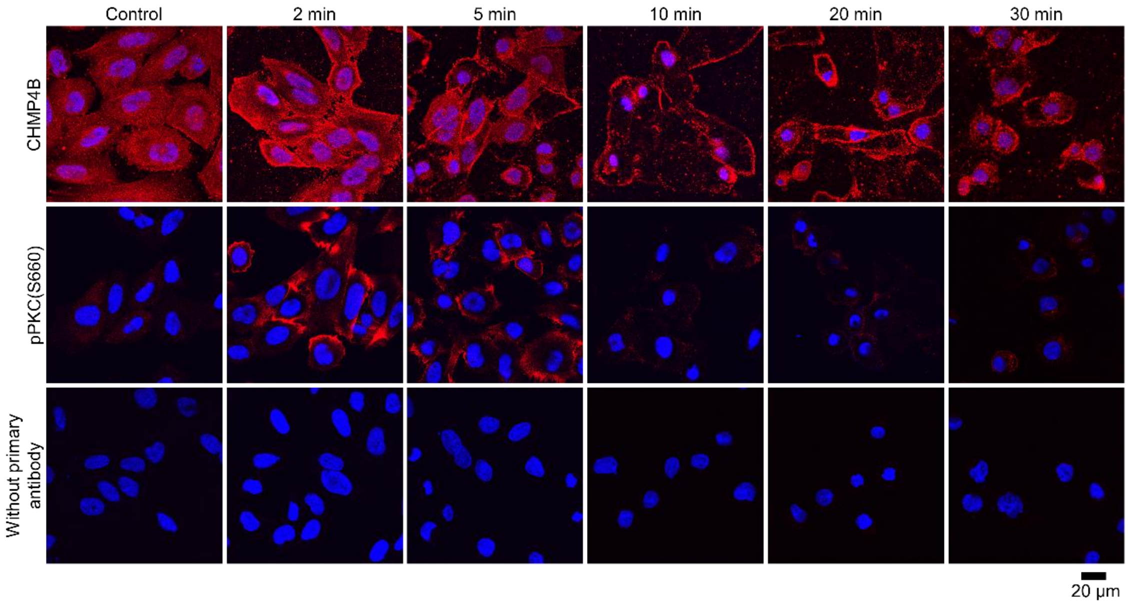

Figure S7. The plasma membrane translocation of CHMP4B and pPKC(S660) of Saos2 with the treatment of 1P (100 μM) at different time points. The control group without the treatment of primary antibody. Red color, CHMP4B or phosphorylated PKC(S660); Blue color, nucleus.

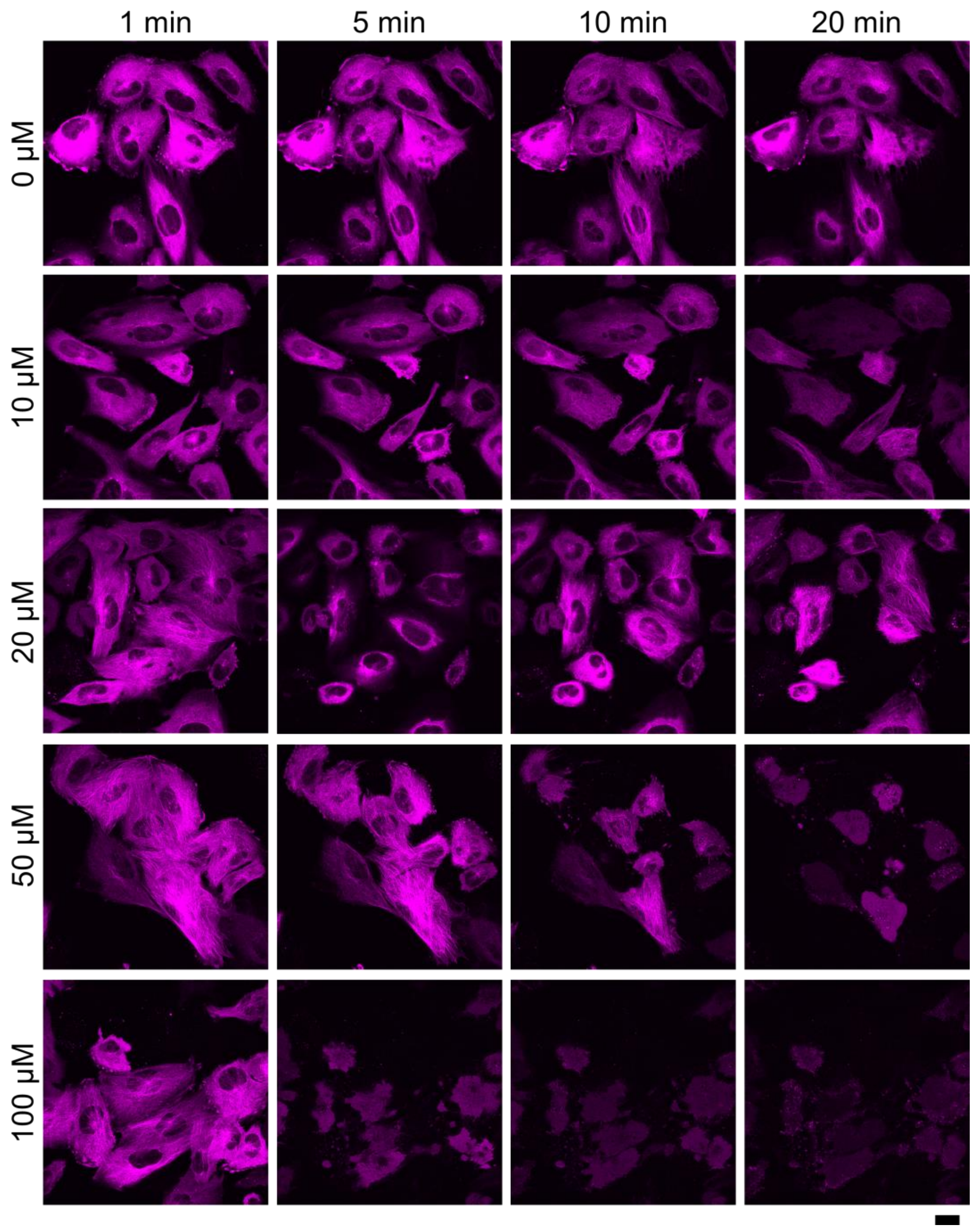

Figure S8. The disruption of tubulin structures of α-tubulin-mCherry transfected Saos2 cells with the treatment of 1P (0, 10, 20, 50, and 100 µM) over 20 minutes. The scale bar is 20 µm.

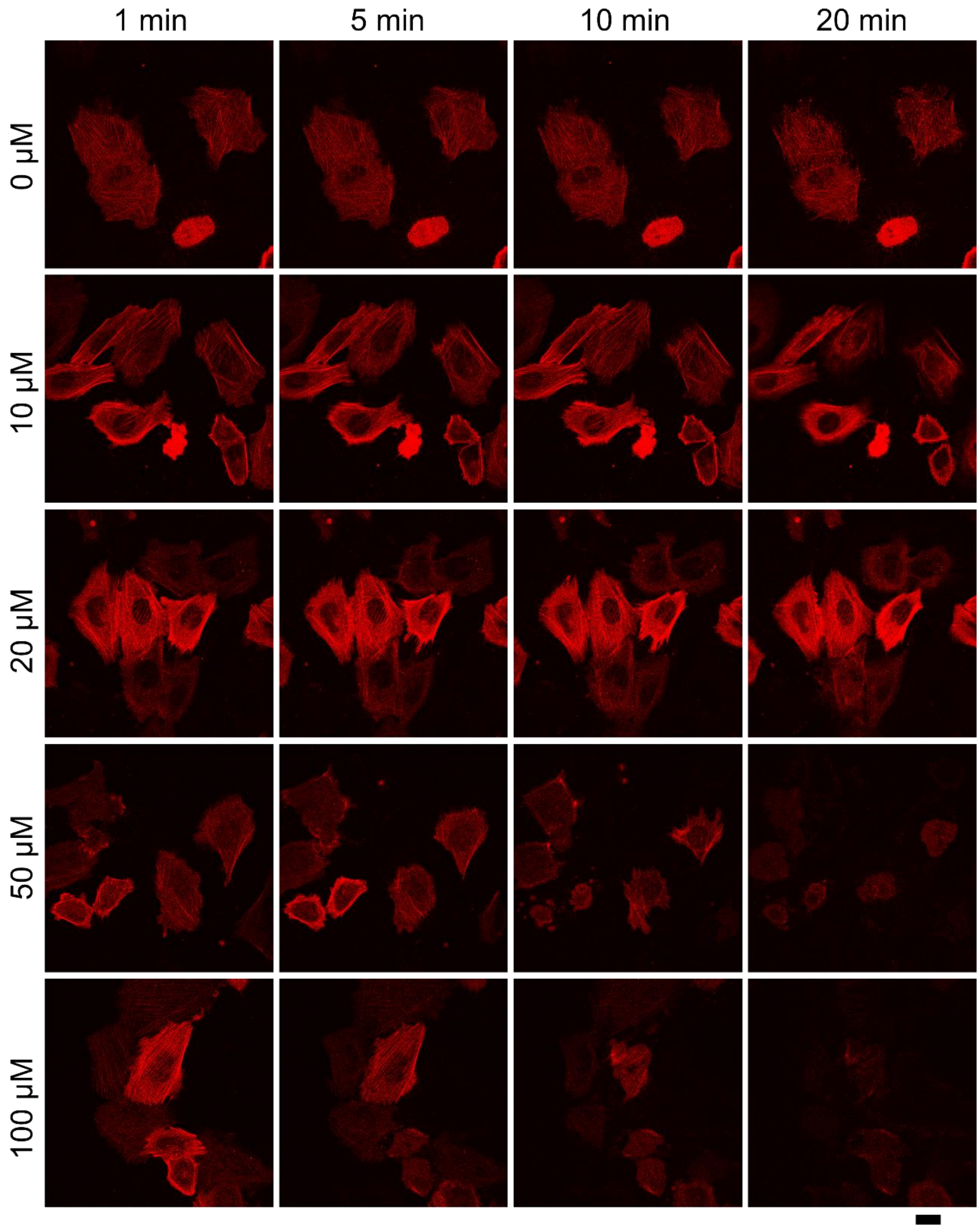

Figure S9. The disruption of F-actin structures of pPAM-β-actin-mCherry transfected Saos2 cells with the treatment of 1P (0, 10, 20, 50, and 100 μM) over 20 minutes. The scale bar is 20 μm.

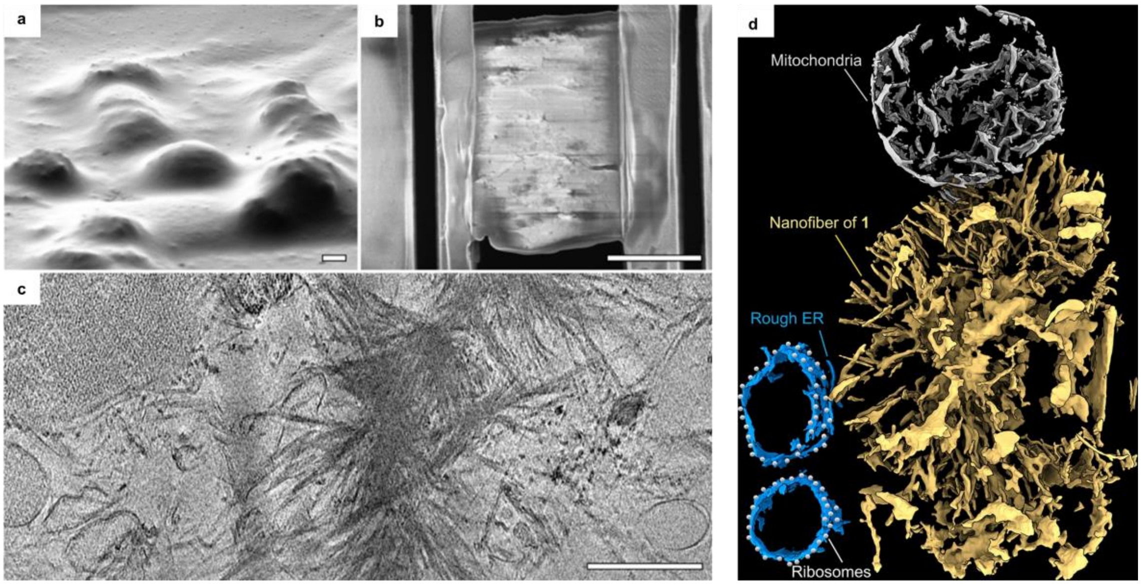

Figure S10. a. SEM image of several Saos2 cells, incubated by 1P (200 μM), on an EM grid. Scale bar 5 μm. b. A SEM image of the Saos2 cell after cryo-FIB milling. c. A cryo-ET image shows an extensive bundle of nanofibers inside the Saos2 cell. Scale bar 100 nm.

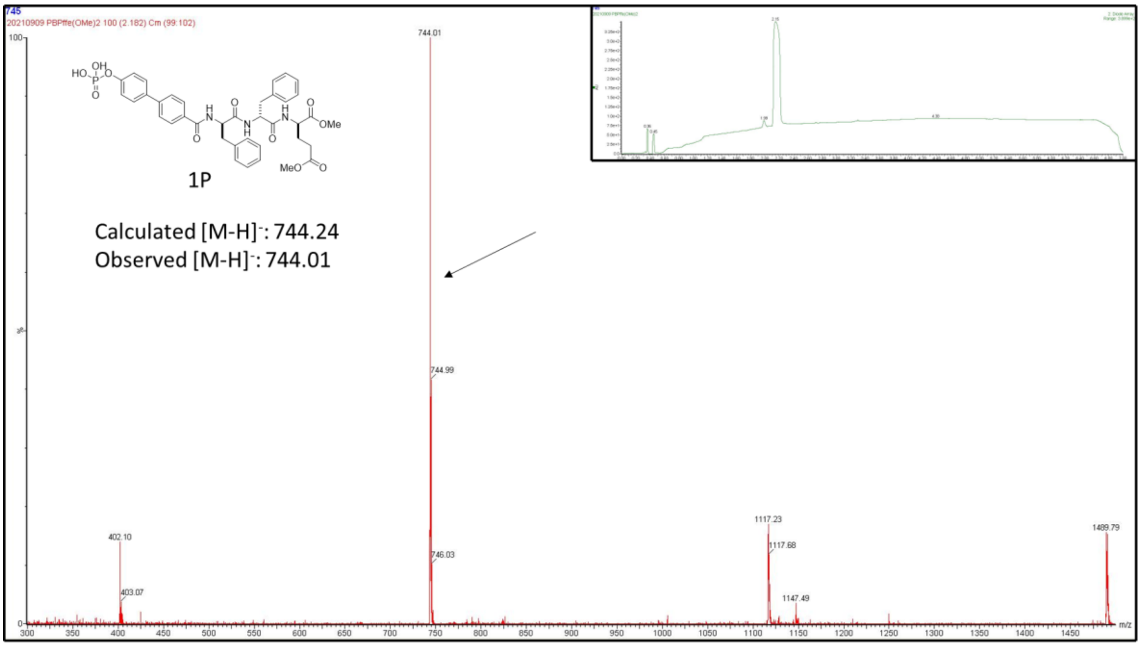

Figure S11. The LC spectrum of 1P (inset) and its corresponding mass spectrum.

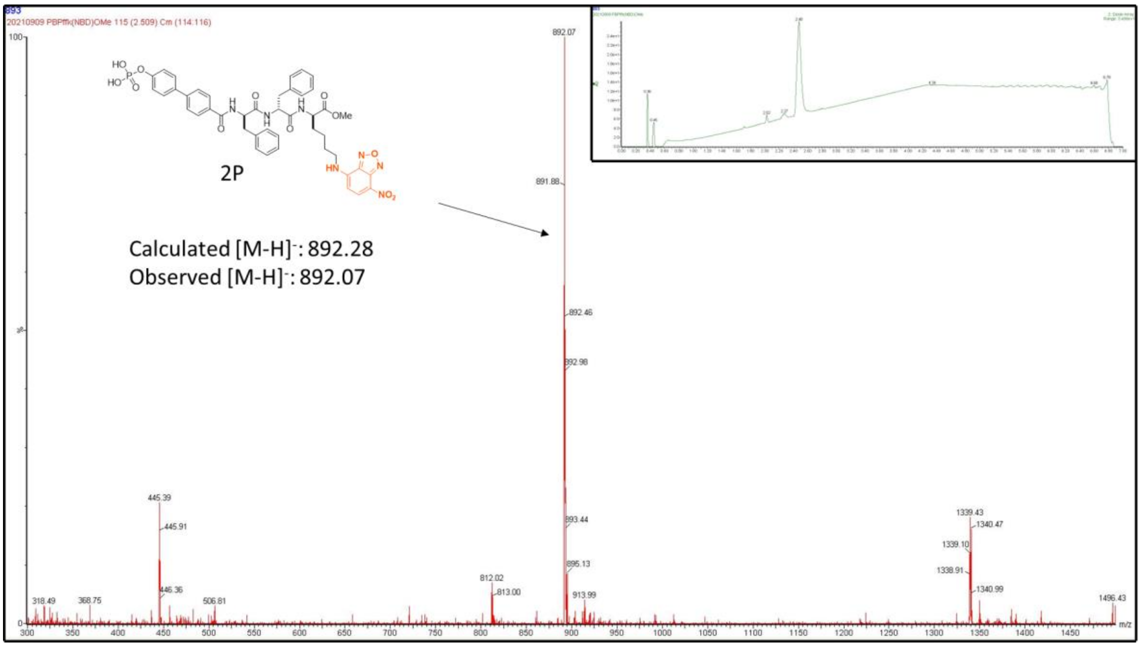

Figure S12. The LC spectrum of 2P (inset) and its corresponding mass spectrum.

Table S1. Cryo-EM and refinement statistics

Parameters	Class1	Class2	Class3	Class4
**Data collection and processing**				
Voltage (kV)	300	300	300	300
Electron dose (e^{-} Å^{-2})	48	48	48	48
Pixel size (Å)	1.08	1.08	1.08	1.08
Particle images (n)	945,036	621,237	472,491	472,586
Shift (pixel)	12.5	12.5	12.5	12.5
**Helical symmetry**				
Point group	C1	C1	C1	C1
Helical rise (Å)	0.269	0.285	0.305	0.329
Helical twist (°)	-19.906	-21.083	22.590	24.095
**Map resolution (Å)**				
Map:map FSC (0.143)	2.9	2.8	2.6	2.6
Model:map FSC (0.5)	3.6	4.0	4.0	3.4
d_{99}	3.1	2.8	3.0	2.9
**Refinement and Model validation**				
Clashscore	7.19	19.42	7.19	25.7
Ramachandran Favored* (%)	100%	100%	100%	100%
RSCC	0.75	0.76	0.73	0.73
**Deposition ID**				
PDB (model)	9NXZ	9NWR	9NWV	9NY0
EMDB (map)	EMD-49913	EMD-49891	EMD-49895	EMD-49914

Video S1. The morphological change of tubulin of Saos2 treated with 50 μM 1P for 30 minutes.

Video S2. The morphological change of actin of Saos2 treated with 50 μM 1P for 30 minutes.

Video S3. Time-resolved cryo-tomograms and segmentation showing the interaction of 1P (100 μM) with the plasma membrane of Saos2 at 2 minutes.

Video S4. Time-resolved cryo-tomograms and segmentation showing the interaction of 1P (100 μM) with the plasma membrane of Saos2 at 5 minutes.

Video S5. Time-resolved cryo-tomograms and segmentation showing the interaction of 1P (100 μM) with the plasma membrane of Saos2 at 8 minutes.

REFERENCE

1. Rohou, A. & Grigorieff, N. CTFFIND4: Fast and accurate defocus estimation from electron micrographs. *J Struct Biol* **192**, 216-221 (2015).
2. Zheng, S.Q. et al. MotionCor2: anisotropic correction of beam-induced motion for improved cryo-electron microscopy. *Nat Methods* **14**, 331-332 (2017).
3. Punjani, A., Zhang, H. & Fleet, D.J. Non-uniform refinement: adaptive regularization improves single-particle cryo-EM reconstruction. *Nat Methods* **17**, 1214-1221 (2020).
4. Wang, F., Gnewou, O., Solemanifar, A., Conticello, V.P. & Egelman, E.H. Cryo-EM of Helical Polymers. *Chem Rev* **122**, 14055-14065 (2022).
5. Emsley, P. & Cowtan, K. Coot: model-building tools for molecular graphics. *Acta Crystallogr D Biol Crystallogr* **60**, 2126-2132 (2004).
6. Afonine, P.V. et al. Real-space refinement in PHENIX for cryo-EM and crystallography. *Acta Crystallogr D Struct Biol* **74**, 531-544 (2018).
7. Xu, A. & Xu, C. FastTomo: A SerialEM Script for Collecting Electron Tomography Data. *bioRxiv*, 2021.2003.2016.435675 (2021).
8. Kremer, J.R., Mastronarde, D.N. & McIntosh, J.R. Computer Visualization of Three-Dimensional Image Data Using IMOD. *Journal of Structural Biology* **116**, 71-76 (1996).
9. Mastronarde, D.N. & Held, S.R. Automated tilt series alignment and tomographic reconstruction in IMOD. *Journal of Structural Biology* **197**, 102-113 (2017).
